# Overview of RAG-based and LLM-based approaches to personalization in healthcare AI applications

**DOI:** 10.3389/frai.2026.1785766

**Published:** 2026-07-29

**Authors:** Manal Althobaiti, Minhee Jun

**Affiliations:** Electrical Engineering and Computer Science, The Catholic University of America, Washington, DC, United States

**Keywords:** evaluation metrics, healthcare AI, large language models, personalization, retrieval-augmented generation, trustworthy AI

## Abstract

Recent advances in Large Language Models (LLMs), driven by transformer architectures such as Generative Pre-Trained Transformer (GPT), are opening new frontiers in healthcare Artificial Intelligence (AI) by enabling clinically relevant interactions between patients and clinicians. Yet persistent challenges—including limited real-time knowledge access, safety concerns and insufficient patient-centered contextualization—indicate that current systems often fall short in delivering efficient and reliably personalized responses (Li et al., 2025; Cascella et al., 2023). To address these gaps, Retrieval-Augmented Generation (RAG) can couple LLMs with external knowledge sources at inference time, producing responses that are more grounded, up-to-date, and tailored to individual patient needs. This systematic review examines how personalization is operationalized in LLM-only and RAG-enhanced healthcare AI systems. We searched multiple scholarly sources and included 20 studies in the final qualitative synthesis. We compare personalization strategies, evaluation practices, and trustworthiness challenges, with emphasis on healthcare-specific issues such as privacy, safety, and clinically meaningful context integration. We analyze how current evaluation frameworks assess these systems and identify limitations in their ability to reflect clinically meaningful performance. We further include a brief MedQuAD-based illustrative case study to highlight limitations of current evaluation metrics, demonstrating that strong benchmark performance does not necessarily correspond to clinically reliable or deployment-ready behavior. The review finds that personalization remains inconsistently defined and weakly evaluated in healthcare AI, with most systems implementing retrieval-grounded adaptation rather than true patient-specific personalization. Furthermore, relatively few studies directly evaluate hallucination, patient safety, clinician validation, or deployment robustness, revealing a critical gap between commonly reported performance metrics and the requirements of clinically reliable, safe, and real-world deployable decision-support systems.

## 1 Introduction

Generative artificial intelligence (AI), in particular LLMs have recently come to the fore across many application domains for its natural, user-friendly interactions. In healthcare, LLMs hold promise for analyzing patient symptoms through conversational interviews, summarizing histories, assisting with documentation, and translating complex clinical concepts into accessible language for patients and providers. However, there is a main limitation: a lack of robust personalization. In practice, most of these applications powered by LLMs produce generic responses that do not adequately reflect an individual patient’s history, risk factors, or context. Healthcare is inherently individualized, and its applications should be tailored to a person’s longitudinal medical history, preferences, genetics, and social and environmental context. Without mechanisms to incorporate these factors, AI systems risk producing outputs that are insufficiently precise or clinically meaningful. Trustworthy personalization further depends on grounding responses in clinical knowledge and records reviewed by medical professionals; when such sources inform model outputs, adaptation can be accelerated, and reliability improved. Consequently, information retrieval becomes essential for individualized care by enabling access to relevant personal health data at the point of need.

**Figure 1:**
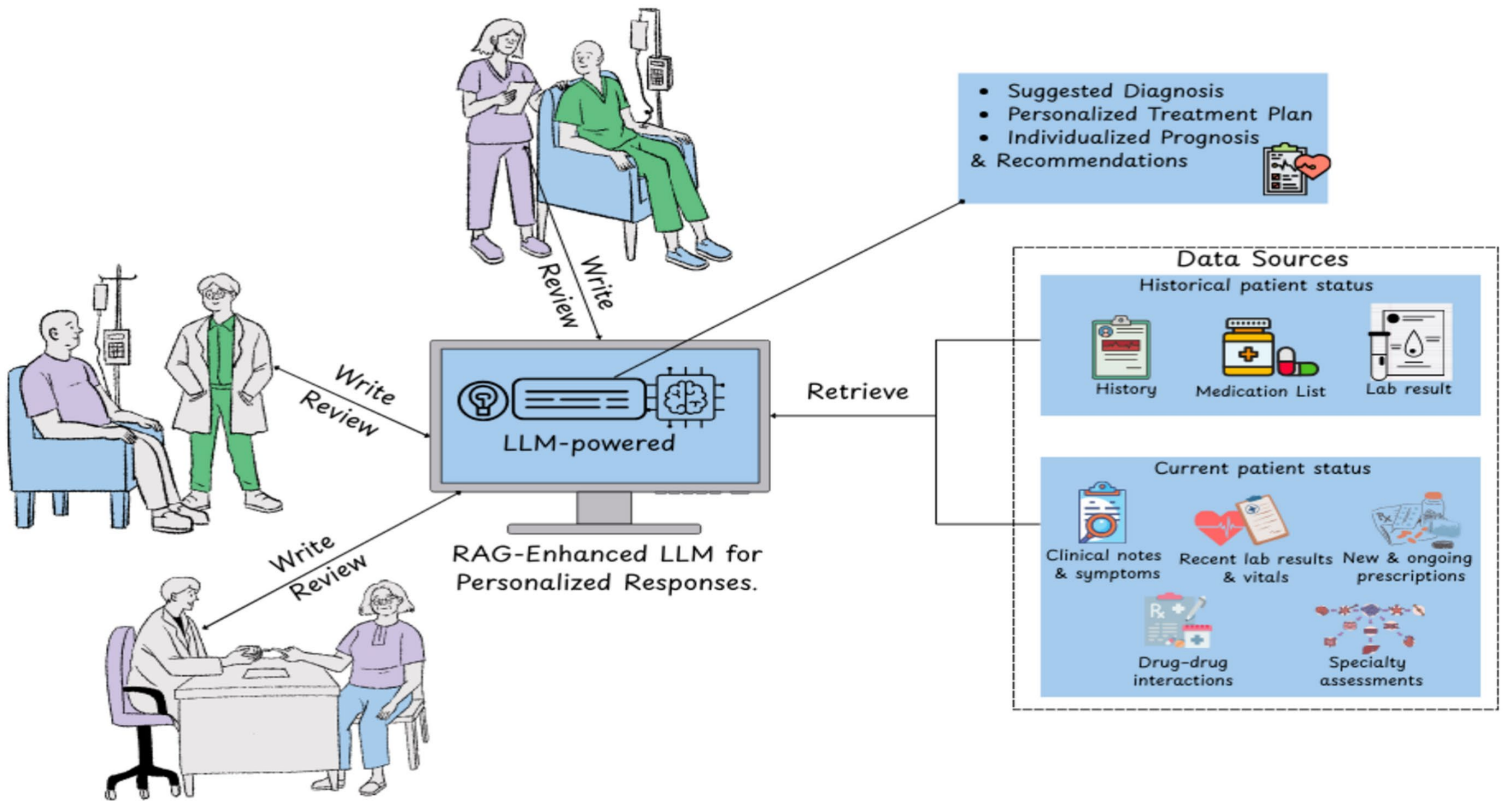
Integrating RAG with LLMs for personalized clinical decision support.

To address this gap, researchers are increasingly turning to personalization techniques powered by LLMs and RAG. Standard LLMs learn from fixed pretraining corpora and do not automatically incorporate new documents or real-time evidence; their knowledge is effectively frozen at training time. RAG complements LLMs by first retrieving salient information from large external stores and then conditioning generation on the retrieved context. Integrating RAG with LLMs enables inference-time access to external knowledge—including patient-specific artifacts (e.g., notes, lab trends) and electronic health records—and Figure 1 shows how incorporating patient historical and current data at inference-time enables the model to generate more personalized responses that are contextually grounded and temporally up to date. By bridging LLMs with real-time, trustworthy retrieval, RAG opens new avenues for transforming unstructured clinical text into actionable insights and tailoring outputs to user-specific traits, thereby improving engagement and satisfaction in sensitive domains such as healthcare. This combined paradigm supports natural, real-time interactions while enhancing a system’s ability to interpret, contextualize, and anticipate user needs (Salemi et al., 2023), enabling personalized systems that are distinct from generic counterparts.

Despite the growing number of surveys on LLM-only and RAG-enabled frameworks in healthcare AI, existing reviews predominantly examine model architectures, methodological designs, evaluation approaches, and ethical considerations in isolation. Comparative synthesis remains limited, particularly with respect to personalization mechanisms, retrieval–generation trade-offs, and evaluation practices across LLM-only and RAG-enabled systems. While clinically validated decision-support systems such as UpToDate rely on curated, structured, and continuously validated medical knowledge, current LLM- and RAG-based systems operate primarily on unstructured retrieval pipelines without formal validation workflows. This distinction motivates a more critical examination of evaluation practices and their ability to reflect clinical reliability, safety, and deployment readiness. Therefore, the goal of this survey is to provide a systematic and comprehensive review of personalization strategies in healthcare AI applications based on LLM-only and RAG-enabled frameworks. This survey synthesizes recent advances by analyzing 20 papers published over the last 5 years, with an emphasis on comparative analysis of methodological designs, retrieval–generation trade-offs, and evaluation practices, as well as practical constraints encountered in real-world healthcare settings. In particular, the survey highlights limitations in current evaluation frameworks and their ability to reflect clinical reliability and deployment readiness. To clarify the scope and novelty of this survey—through a unified comparative analysis with an explicit evaluation-centered perspective, experimental validation, and a future research roadmap—relative to prior work, Table 1 compares representative existing surveys (Zhang et al., 2025; De Pinho Souza et al., 2025; Li et al., 2025) with respect to their coverage of LLM-only and RAG-based personalization strategies, evaluation metrics, simulation experiments, and roadmaps for trustworthy, reliable, and regulation-compliant healthcare AI.

**Table 1:** Comparison between previous studies and our study.

Reference	Personalization as central theme	Evaluation synthesis	Empirical case study	Trustworthiness discussion	Healthcare focus
Zhang et al. (2025)	✓	✓	✗	✗	✗
Li et al. (2025)	✓	✓	✗	✓	✗
De Pinho Souza et al. (2025)	✓	✓	✗	✓	✓
This Study	✓	✓	✓	✓	✓

This paper’s contributions are as follows:

- We provide a focused systematic review of personalization strategies in LLM-only and RAG-based healthcare AI.
- We organize the literature using a taxonomy of parametric, retrieval-based, and hybrid personalization strategies, highlighting their strengths, limitations, and trade-offs across multiple healthcare-related tasks, including considerations of privacy, latency, and faithfulness.
- We include a brief illustrative MedQuAD-based case study to show why current evaluation metrics can be misleading when interpreted as evidence of true personalization, and to demonstrate the gap between benchmark performance and clinically meaningful reliability.
- We provide a roadmap for future research toward trustworthy, reliable, and regulation-compliant healthcare AI systems, emphasizing the need for evaluation frameworks that incorporate clinical validation, safety, and deployment constraints, particularly focus on personalization at the intersection of LLMs and RAG.

This paper is organized as follows. Section 2 presents the review methodology, including the search strategy, inclusion and exclusion criteria, quality assessment, and study selection process. Section 3 reviews the background and key concepts related to large language models (LLMs) and retrieval-augmented generation (RAG). Section 4 surveys existing personalization approaches and identifies open gaps in current studies and evaluation frameworks in relation to clinical applicability. Section 5 presents the experimental setup and results, examining the limitations of current evaluation metrics. Section 6 provides the discussion. Section 7 outlines future research directions. Finally, Section 8 concludes the paper.

## 2 Review methodology

Because LLM and RAG techniques are evolving rapidly, while journal publication cycles remain lengthy, researchers often disseminate recent findings before formal publication. However, this review prioritizes peer-reviewed studies published in conference proceedings and journals. Our study selection began with a comprehensive search across IEEE Xplore, the ACM Digital Library, PubMed, and MDPI, supplemented by records identified through Google Scholar and Semantic Scholar. To ensure the credibility, rigor, and transparency of the cited literature, we adopted a classification-based review methodology guided by established principles of systematic literature review. Specifically, we followed well-established guidelines in information systems and software engineering as proposed by (Wohlin, 2014; Okoli and Schabram, 2015), which provide a systematic framework for collecting, screening, and evaluating studies from multiple scholarly databases on personalization strategies using LLMs and RAG frameworks in AI applications, along with their limitations, thereby ensuring a clear and replicable review of existing research.

### 2.1 The search strategy

We defined specific search keywords to capture a broad range of relevant studies that met our inclusion criteria. These keywords were related to personalization strategies in AI healthcare applications, particularly those involving LLMs and Retrieval-based. An initial exploration of existing literature helped identify frequently used terms in this research domain. Accordingly, the keywords employed included (*“LLM-based healthcare systems,” “retrieval-augmented generation in healthcare,” “retriever-based healthcare systems,” “LLM-based clinical applications,” “conversational AI in healthcare,” “healthcare chatbots,” “medical conversational agents,” and “personalized healthcare AI.”*) Moreover, inclusion and exclusion criteria were defined to filter the search results (see Section 2.2). The quality assessment measures were introduced in Section 2.3 and 2.4 to evaluate the robustness of the selected literature.

### 2.2 Inclusion and exclusion criteria

We defined five inclusion criteria and four exclusion criteria, as summarized in Tables 2, 3. During the data retrieval stage from the selected databases, we limited the publication period to 2022–2026 and selected only English-language, peer-review and conference papers available in the chosen database. All duplicate papers were removed. In the initial screening phase, titles and abstracts were reviewed to identify studies relevant to LLM-based and retriever-based personalization AI healthcare applications. Studies that fell outside the scope of personalization—such as those focusing on unrelated to LLM and retrieval techniques—were excluded. Additionally, papers lacking a clear methodological framework or those that were inaccessible were omitted from the review. These systematic filtering process ensured that only methodologically sound and relevant studies were included for further analysis.

**Table 2:** Inclusion criteria.

Criteria	Description
1	The studies were published between 2022 and 2026.
2	The studies were published in conferences, journals or selecting highly cited literature
3	The studies are written in English.
4	The full text is available and published.
5	The papers must relate to LLM-based and retrieval-based personalization in healthcare

**Table 3:** Exclusion criteria.

Criteria	Description
1	Studies which are not related to the topic or healthcare field or out of range dates.
2	Studies which are not related to hybrid personalization and retrieval-based personalization.
3	Studies with no clear information or methodology.
4	Studies with preprint version or not published.

### 2.3 Quality assessment

To ensure that all selected studies were methodologically sound and relevant, we performed a quality assessment as summarized in Table 4 (a). Each full-text paper was evaluated against five predefined criteria, receiving a score of 1 (yes) or 0 (no) for each criterion. Only studies achieving a total score of 5 were included in the final review [see Table 4 (b) and (c)]. Consequently, the selected papers focused on text-based hybrid personalization and retriever-based personalization, employed generation and/or retrieval, reported a clear methodological framework, specified the datasets used, and presented measurable evaluation results.

**Table 4:** Quality assessment criteria and results (a)–(c).

(a) Quality assessment criteria		
Criteria	Description	Evidence indicator
E1	Does the study focus on text-based information retrieval using LLMs, RAG or hybrid retrieval-generation in personalization healthcare?	Relevance to text-based personalization or information retrieval in healthcare.
E2	Does the study apply AI-based retrieval or generation within its proposed methodology?	Utilizes fine-tuned LLMs, RAG, prompt engineering, or hybrid retrieval-generation approaches.
E3	Is the proposed method clearly presented and sufficiently reproducible?	Provides methodological details such as architecture, workflow, model configuration and retrieval strategy.
E4	Does the study include or cite datasets used for model training, evaluation, or validation?	Clearly specifies dataset source, type (e.g., EHR, clinical notes, QA datasets), preprocessing steps, and accessibility.
E5	Are the experimental results and evaluation procedures reported with sufficient detail? Does the study report meaningful metrics?	Includes quantitative results, evaluation metrics (e.g., precision, recall, faithfulness, hallucination rate, clinical relevance), baseline comparisons and validation procedures.

(b) Quality assessment result (1)	
Quality criterion	Studies meeting criterion
Clear healthcare task	20/20
LLM or RAG method described	20/20
Dataset specified	20/20
Evaluation metrics reported	20/20
Personalization relevance	20/20

(c) Quality assessment result (2)	
Quality score	Number of studies
5/5	20
4/5	0
3/5 or lower	0

### 2.4 Study selection procedure

**Figure 2:**
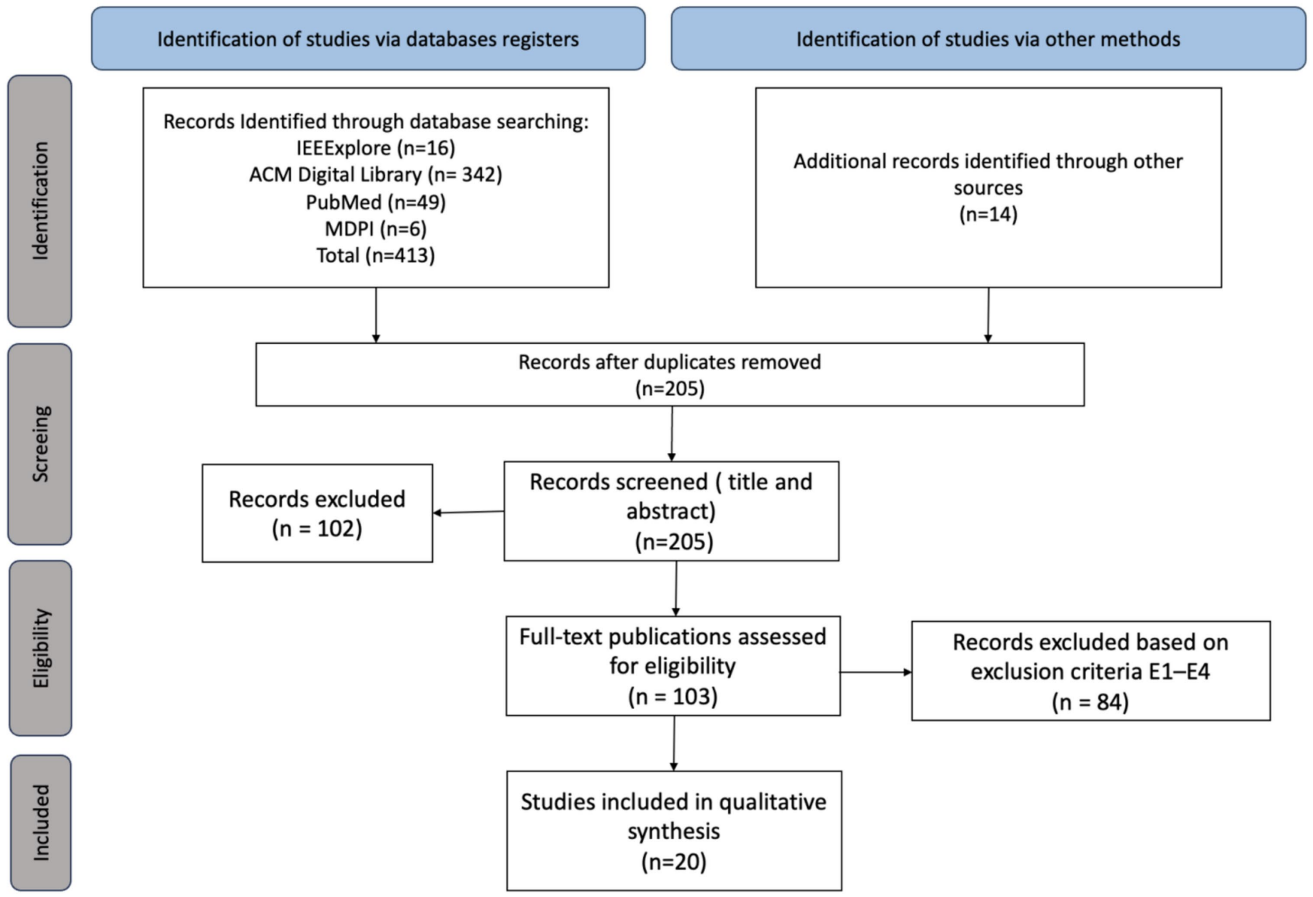
The phases of the study selection process.

The study selection process followed PRISMA guidelines for transparent identification, screening, eligibility assessment, and inclusion of studies. First, the primary author independently screened the titles, abstracts, and full texts of the retrieved articles based on the predefined inclusion and exclusion criteria. Initial screening and data extraction were conducted by the first author, while eligibility decisions and synthesis categories were reviewed and validated through consultation with the supervising co-author to ensure consistency, relevance, and methodological rigor. Any uncertainties regarding study eligibility were discussed and resolved through consultation. Searches conducted between 2022 and 2026 yielded a total of 427 records, including 413 records identified through academic database searching and 14 additional records identified through other sources. After removing duplicates, 205 unique records remained. Titles and abstracts were screened according to the inclusion and exclusion criteria described in Section 2.2, resulting in 103 potentially eligible records, while 102 records were excluded for not meeting the predefined criteria. Most exclusions were due to irrelevance to the personalization scope, lack of a clear methodological framework, or non-healthcare and non-text-based focus. Subsequently, 103 full-text articles were assessed for eligibility, and 84 articles were excluded based on exclusion criteria E1–E4. Finally, 20 studies were included in the qualitative synthesis. Figure 2 illustrates the study selection process and summarizes the final set of included studies. Formal inter-rater agreement metrics, such as Cohen’s Kappa, were not computed, which we acknowledge as a limitation of the review process.

### 2.5 Screening workflow

**Figure 3:**
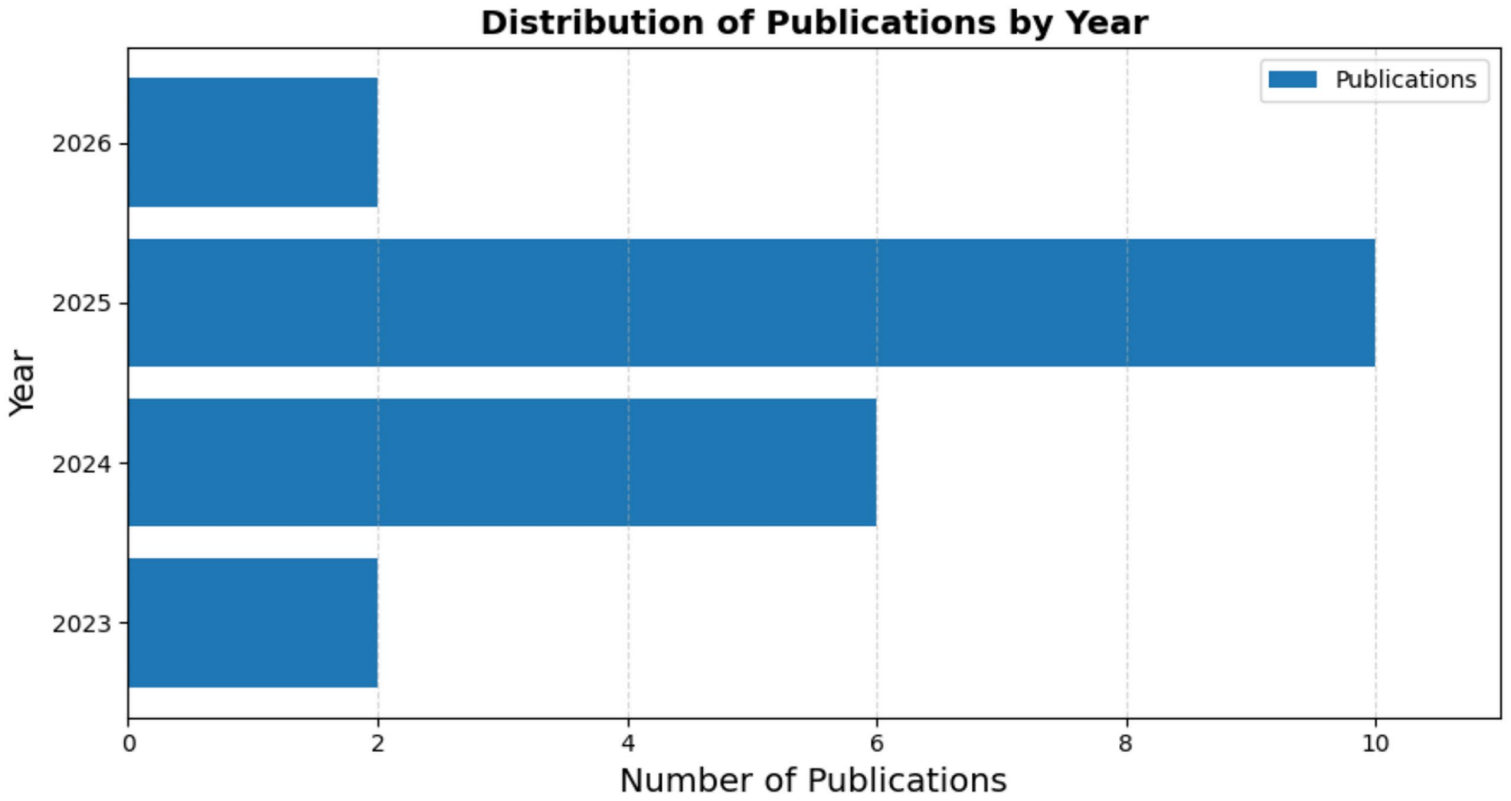
Distribution of publications by year.

The screening and organization process was supported by standard review tools. The primary author used EndNote to manage records and remove duplicate studies. Rayyan was used for title and abstract screening based on the predefined inclusion and exclusion criteria, while full-text assessment and data extraction were conducted by the first author. Eligibility decisions and synthesis categories were reviewed with the supervising co-author to ensure consistency and methodological rigor. Finally, Microsoft Excel was employed to summarize, organize, and synthesize the final findings. As shown in Figure 3, the selected studies provide an increasing publication trend, particularly from 2024 to 2025, reflecting the growing research interest in this area.

### 2.6 Review limitations

Although this review followed PRISMA guidelines and applied a structured screening methodology, several limitations should be acknowledged. First, the review was restricted to studies published in English between 2022 and 2026, which may have excluded relevant research in other languages or outside this time range. Second, the selected databases primarily included peer-reviewed journal and conference papers and where multiple versions of a study existed, the most recent peer-reviewed publication was selected to ensure scholarly rigor. In addition, the initial screening and data extraction were primarily conducted by the first author, while eligibility decisions and synthesis categories were reviewed with the supervising co-author rather than through full dual-independent screening. Although this process helped ensure consistency and methodological rigor, some degree of selection bias may remain. Furthermore, because healthcare personalization using LLMs and RAG is an emerging field, the available literature remains limited and highly heterogeneous in terms of datasets, evaluation methods, and personalization strategies, which may affect direct comparison across studies. To support this comparative analysis, it is first necessary to briefly outline the technical foundations of LLM-based and retrieval-based personalization. The following background section is intentionally concise and limited to concepts directly relevant to patient-centered contextualization.

## 3 Background

### 3.1 Large language model

**Figure 4:**
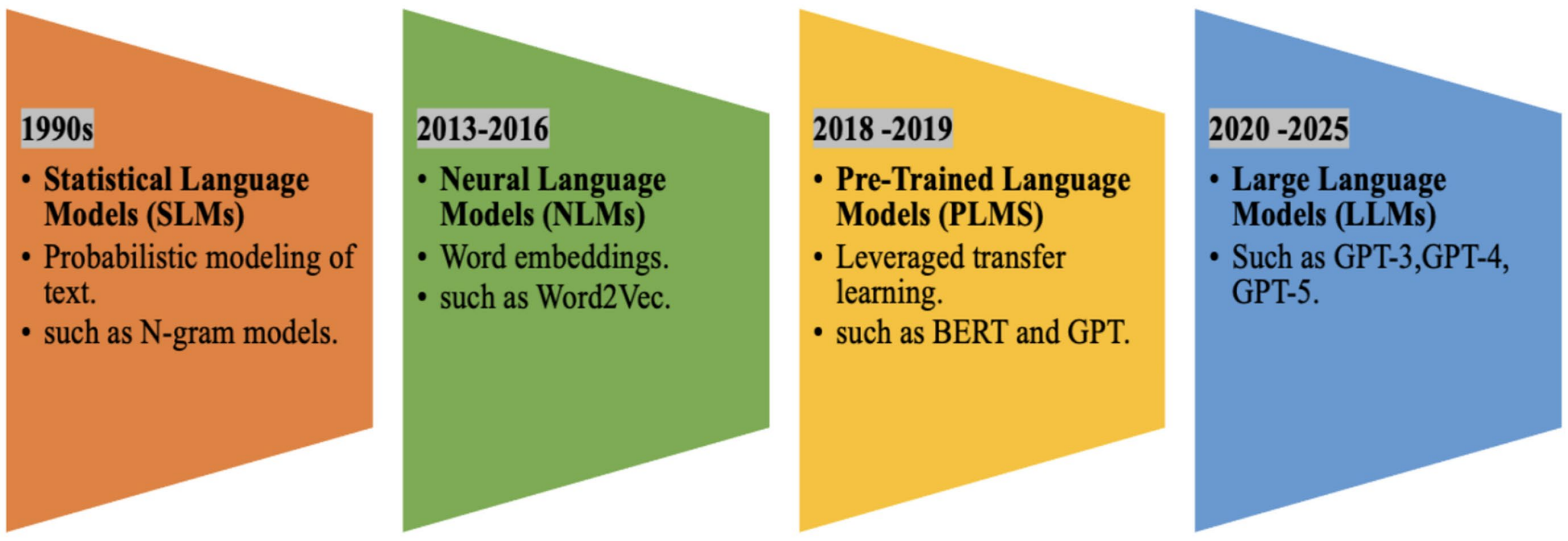
Historical development of LLMs.

LLMs are deep neural networks—predominantly based on transformer architectures—that encode vast amounts of linguistic and world knowledge in billions of parameters (e.g., the GPT-4/5 families). Trained using next-token prediction on large-scale corpora (curated datasets of text or speech), LLMs acquire broad capabilities in text generation, comprehension, summarization, and information extraction. In healthcare, these capabilities enable human-like language production grounded in the statistical regularities learned during pretraining (Brown et al., 2020; Wang et al., 2025). Figure 4 illustrates the evolution of language modeling, progressing from statistical language models (SLMs) based on n-gram methods, to neural language models (NLMs), then pre-trained language models (PLMs) such as BERT and GPT, and finally to modern large language models (LLMs) including GPT-2, GPT-3, ChatGPT, and GPT-4. It highlights how increasing model scale and transformer-based architectures improved contextual understanding, reasoning, and generalization across Natural Language Processing (NLP) tasks. LLM behavior is guided at inference time through prompts, which consist of textual inputs and instructions that condition the model’s responses. Effective prompt design—commonly referred to as prompt engineering—can significantly influence output quality, consistency, and reliability, and serve as a complementary approach to model adaptation. Adaptation techniques range from lightweight methods, such as instruction prompting and retrieval conditioning, to full fine-tuning, where a pre-trained model is updated using curated datasets to improve task performance, reduce hallucination or repetition, and better align outputs with human preferences (Zhang et al., 2024). Collectively, these advances underpin modern, instruction-following LLMs and lay the foundation for retrieval-augmented approaches such as RAG that ground model outputs in external knowledge sources—an essential capability for building safe, reliable, and personalized healthcare AI systems.

#### 3.1.1 Embeddings

**Figure 5:**
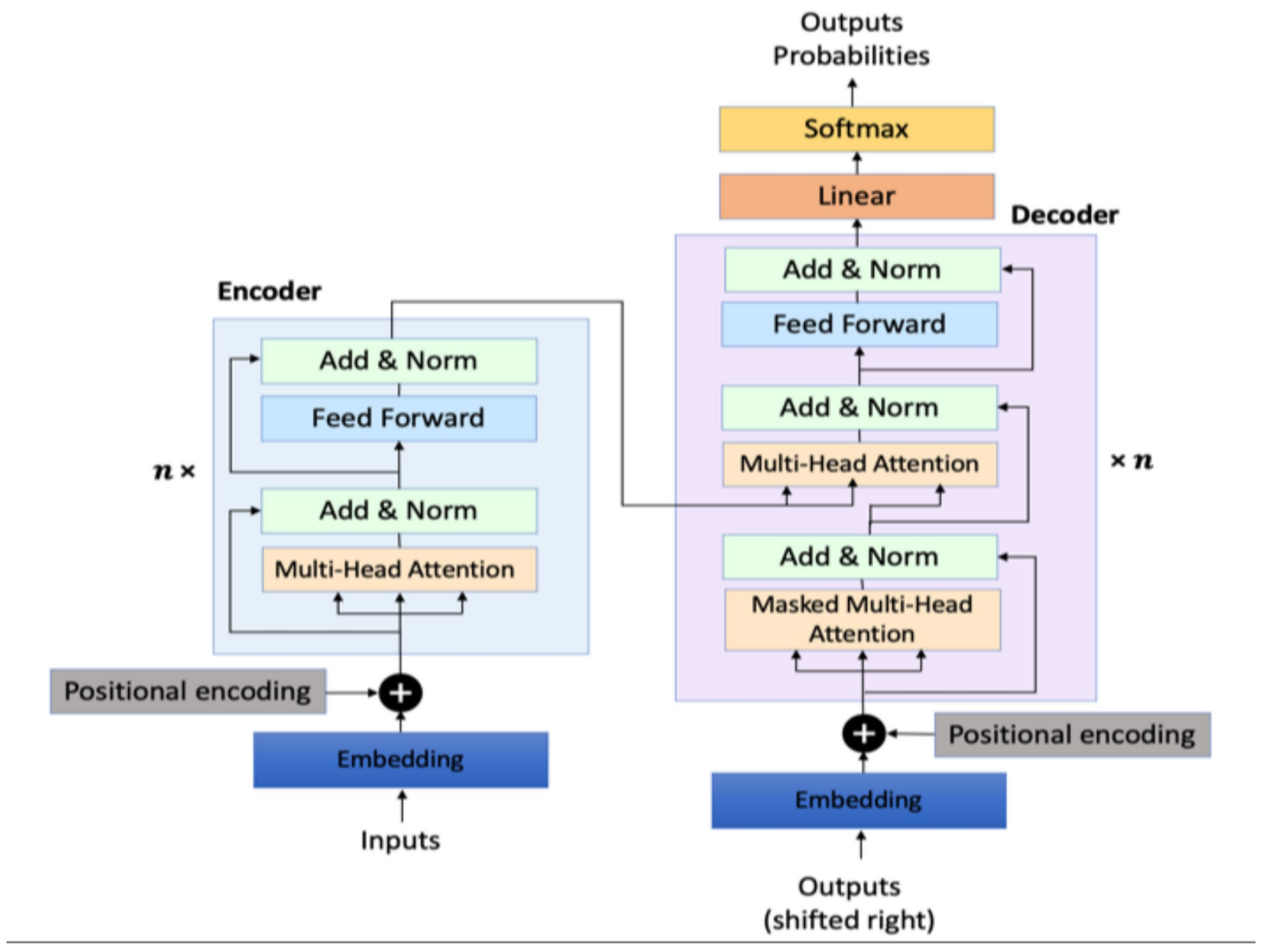
Transformer model (2017).

Vector representations convert text, audio, and images into numerical forms that make analysis more efficient and scalable. In LLMs, words, sentences, and paragraphs are transformed into embeddings, enabling semantic search beyond simple keyword matching. Transformer-based models use self-attention and positional encoding to capture long-range dependencies and contextual meaning. These models are generally grouped into three categories: encoder-only, decoder-only, and encoder–decoder architectures. Decoder-only models such as GPT and Llama are effective for text generation and dialogue, while encoder-only models such as BERT perform well in classification and semantic search. Encoder–decoder models support tasks such as translation and summarization (Devlin et al., 2019). Embeddings represent data as points in an n-dimensional space, where semantically similar items are positioned closer together, improving retrieval efficiency (see Figure 5). A query can be embedded and matched to the nearest document vectors, allowing retrieval based on semantic similarity rather than exact keyword matching. Frameworks proposed in (Feng et al., 2023; Sun et al., 2022) focus on enhancing LLMs with external or structured internal knowledge to reduce hallucinations and irrelevant answers. The Knowledge Card framework (Feng et al., 2023), augments LLMs through plug-in, domain-specific models trained on specialized corpora, allowing relevant and factual knowledge to be integrated during inference. While effective, the approach suffers from limitations: the knowledge cards, initialized with a small model (OPT-1.3B), sometimes produce low-quality or off-topic outputs, indicating the need for larger or better-trained models. The factuality selector tends to favor information-rich domains, reducing accuracy in emerging areas, and the yes/no prompting strategy for LLM self-assessment proves only partially effective due to model overconfidence. The Recitation-Augmented Language Model (RECITE) presented in (Sun et al., 2022), which enables LLMs to internally “recite” stored knowledge prior to answer generation, thereby improving reasoning in knowledge-intensive tasks. However, updating time-sensitive information in such LLM-only approaches requires retraining or fine-tuning on new data. Both approaches emphasize integrating conversational context and explicit knowledge citation to enhance factual accuracy and reliability.

#### 3.1.2 Fine-tuning

**Figure 6:**
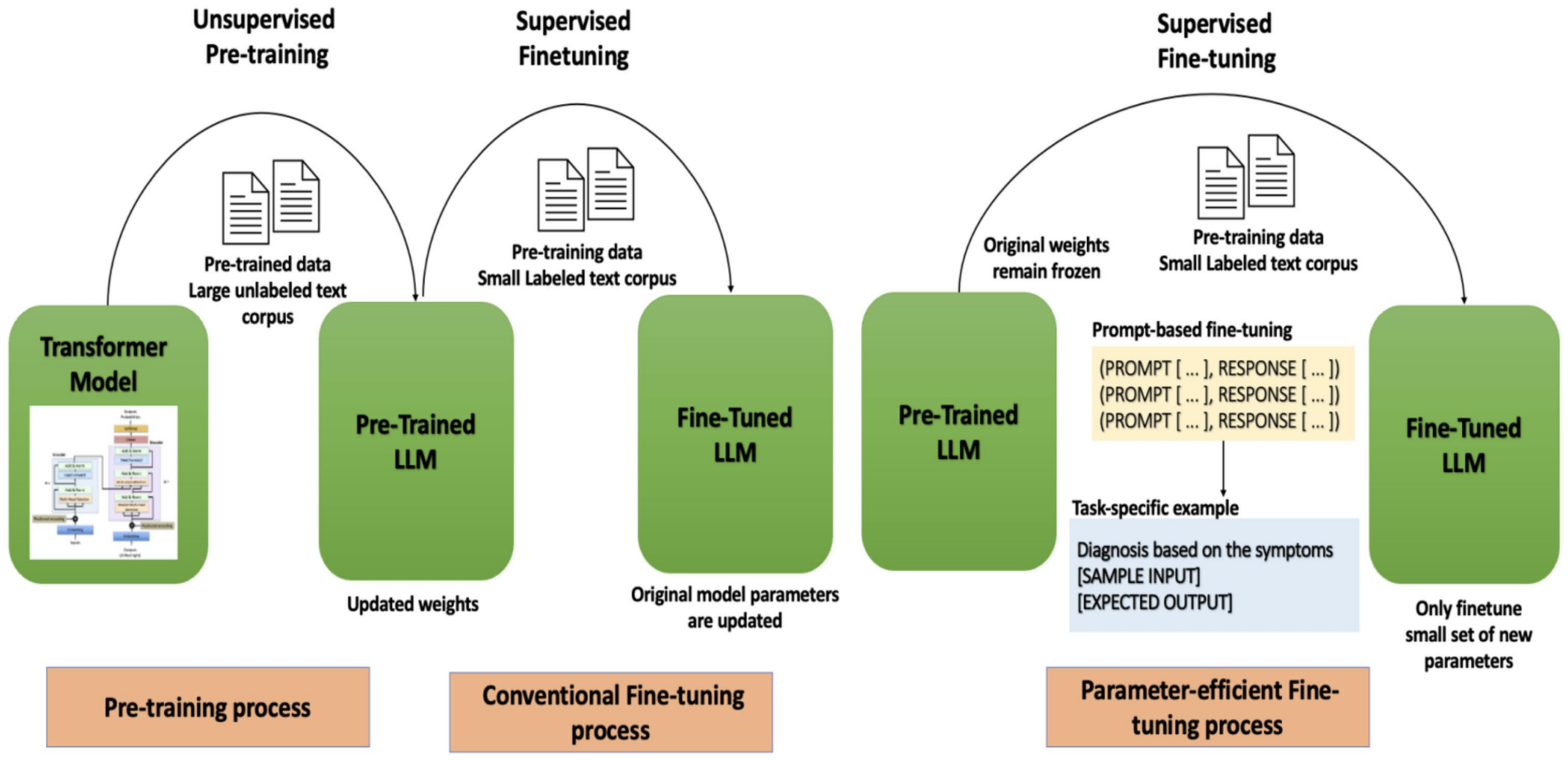
Pre-training and fine-tuning and using prompts to fine-tune LLMs with instruction.

During the computationally intensive pre-training phase, LLMs learn grammar, reasoning, and contextual understanding from large-scale datasets. However, in specialized domains such as healthcare, pre-trained models often struggle with task-specific accuracy and protection of sensitive patient information. These limitations can be addressed through fine-tuning, which adapts pre-trained models using domain-specific data for tasks such as text classification and question answering (see Figure 6). Compared to training from scratch, fine-tuning is more practical and widely adopted.

Two common strategies are feature extraction, where the pre-trained model remains mostly frozen and only task-specific layers are trained, and full fine-tuning, where all parameters are updated. While full fine-tuning often improves performance, it requires significantly higher computational cost. Fine-tuning approaches include supervised fine-tuning (SFT), Reinforcement Learning from Human Feedback (RLHF), and Parameter-Efficient Fine-Tuning (PEFT). PEFT methods such as LoRA update only a small subset of parameters, reducing cost while maintaining strong performance (Wu et al., 2025; Ross et al., 2025).

### 3.2 Retrieval augmented generation

**Figure 7:**
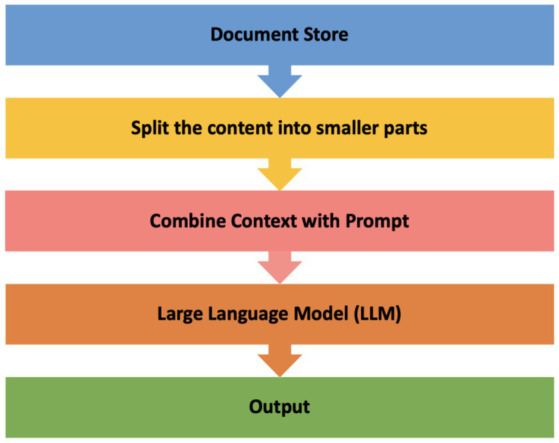
The RAG process.

In contrast to earlier works that concentrated on adapting the language models, personalized conversational information retrieval such as (Mo et al., 2025) identify the required level of personalization for query reformulation. However, evaluation heavily depends on subjective human judgment regarding the usefulness of personal information, with limited attention to privacy risks. In healthcare, combining PEFT with RAG improves retrieval-grounded adaptation by integrating an efficient model with retrieval-based access to external medical knowledge such as patient history or longitudinal EHRs, while keeping sensitive personal data outside global model parameters and supporting stronger privacy protection. This technique uses a vector database to store unstructured text and provide LLMs with external knowledge, allowing models to access updated information and reference sources during response generation. Figure 7 illustrates this process. By integrating RAG with LLMs, the system produces more accurate, relevant, and task-specific outputs.

The workflow includes document preprocessing, chunking, embedding, and indexing in a vector database. When a user submits a query, relevant content is retrieved through similarity-based search and combined with the original prompt before being passed to the LLM. This grounding process helps reduce hallucinations and improves response reliability (Lewis et al., 2020; Yoran et al., 2023). In practice, retrieved evidence enables the model to generate more factual and context-aware answers compared to non-retrieval models. Additionally, (Liu et al., 2025) explores Chain-of-Thought reasoning and LLM-based recommendation generation to enhance reasoning performance; however, this approach is unsuitable for healthcare domains that require real-time domain-specific data handling. Frameworks such as LangGraph and LangChain support modular retrievers, memory, vector stores, and agent-based workflows for scalable healthcare applications (Odofin et al., 2024). However, our findings indicate, integrating patient-specific data into this framework did not always produce meaningful personalization or improved context-aware generation. In addition, handling sensitive medical data within retrieval systems raises significant privacy and security concerns. Recent applications of RAG-based retrieval using vector embeddings (Khan et al., 2024) further show the value of RAG-based retrieval using vector embeddings to improve response accuracy and reduce irrelevant answers in QA. The RAG framework developed by Khan et al. (2024) uses PDF documents and includes data collection, PDF preprocessing, indexing, and response generation, and compares proprietary GPT APIs with open-source LlaMA models. However, during the development stage, it is important to remain aware of moral and societal aspects. This approach is somewhat limited, as it relies on sample dialogues, raising concerns about robustness. In addition, during the evaluation process, the approach depends on the quality of PDF extraction, which may result in incomplete retrieval from documents and lead to inaccurate or misleading information. Across these studies, several key limitations of RAG pipelines emerge. Relevant information is often scattered across multiple sections, yet existing algorithms retrieve only limited chunks, missing important context. They also tend to equate similarity with relevance, which can reduce retrieval accuracy. Also, uniform chunking often ignores document structure, leading to incomplete context extraction. While some of these issues have partial solutions, others remain difficult to address, and continued research is needed to make RAG systems more accurate and robust. Chen et al. (2025) designed a co-modality RAG pipeline for document visual question answering (VQA). It decomposes pages into whole-page images, sub-images, and parsed text, retrieves evidence from both text and image channels, aggregates cross-modal features, and uses a VLM generator to produce answers. Using a co-modality–based RAG instead of a single-modal approach in visual document question answering can lead to increased complexity, as failures in the retrieval and generation pipelines may result in incorrect answers. For combining two personalization strategies for HybridRAG and parameter-efficient fine-tuning (PEFT) (Salemi and Zamani, 2025) evaluates both across seven datasets for user-specific adaptation. RAG retrieves relevant personal context at inference time, while PEFT fine-tunes parameters per user. Results show RAG improves performance by 14.92%, PEFT by 1.07%, and the combined approach by 15.98%. Further, reveals that although PEFT performance improves with larger amounts of user data, RAG proves more effective in cold-start scenarios where user data is limited. Thus, the methodology provides empirical insights into the trade-offs between non-parametric and parametric personalization approaches under privacy-preserving conditions. It highlights that both RAG-based methods and PEFT-based personalization require advances in efficient retrieval pipelines, model compression techniques, and adaptive storage to enable scalable, privacy-preserving, real-world personalization of LLMs.

#### 3.2.1 Vector databases

As opposed to traditional relational databases, unstructured text requires efficient nearest-neighbor search in high-dimensional embedding spaces. Vector databases support this by converting textual data into numerical embeddings, enabling fast storage, indexing, and retrieval for AI models. When integrated with LLMs, vector databases support semantic and multimodal search, where a text query is encoded into a vector and matched with semantically similar content rather than exact keywords. Retrieval can be further improved by combining vector similarity with metadata filtering and hybrid relevance methods such as TF-IDF and BM25. By storing indexed, source-linked embeddings, these systems improve information access, patient engagement, and healthcare efficiency (Salemi et al., 2023; Zhang et al., 2025). However, they remain limited in delivering truly individualized care and raise important privacy and security concerns when handling sensitive medical data.

#### 3.2.2 Knowledge base

**Figure 8:**
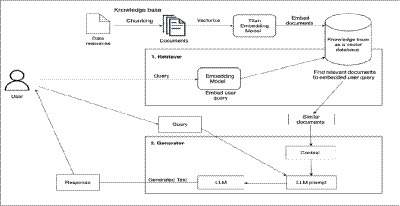
Two types of Information retrieval approaches in integrating knowledge base and LLMs: (1) *Retrieval*: Embed the user query and finds similar documents. (2) *Generator*: Combines the query with the retrieved context and uses the LLM to generate the final response.

A structured repository of domain-specific information is built using a scalable database to store relevant documents and associated metadata. Advanced search capabilities enable rapid and accurate retrieval, functioning similarly to a vector database for efficient indexing and access. This knowledge base serves as an external source that the AI model can query when generating responses, as shown in Figure 8. However, structured knowledge bases may face limitations in retrieval quality and answer accuracy, especially when handling complex, ambiguous, or context-dependent queries.

#### 3.2.3 Knowledge graph

In information retrieval, keyword-based methods such as TF-IDF and BM25 often struggle to capture semantic meaning in unstructured text. Knowledge Graphs address this limitation by organizing entities and their relationships into graph-based structures. By leveraging semantic similarity, synonym alignment, and hierarchical relationships, knowledge-graph-driven retrieval provides more precise, context-aware, and intelligent results than simple keyword matching (Wang et al., 2018). This approach improves retrieval efficiency and accuracy while enabling advanced capabilities such as inference and recommendation. Integrating structured knowledge bases with knowledge graphs is also a promising direction for healthcare personalization, as it enhances response quality while supporting efficiency and privacy preservation. Study (Au et al., 2025) experimentally proposes a personalized graph-based RAG framework evaluated on a personalized benchmark; however, it does not address user privacy and data sensitivity risks and exhibits limited scalability as the user base grows. Recent work has begun exploring patient-specific personalization. For merges knowledge graph retrieval with vector-based retrieval to enhance information extraction from complex domain-specific documents, constructs a knowledge graph to capture structured relationships among entities while also building a dense vector index for unstructured text (Sarmah et al., 2024) During inference, HybridRAG retrieves relevant information from both sources and fuses them to guide a large language model in generating more accurate and context-aware responses. Prahlad et al. (2025) evaluate LLM-based RAG system combined with knowledge graphs to improve accuracy and response time. Although sensitive user data is stored locally to prevent direct exposure to cloud-based LLMs, the LLM provider may still access aggregated user information, raising privacy concerns. This integrated approach outperforms traditional VectorRAG and GraphRAG methods in retrieval precision, response relevance, and factual consistency. This integrated approach may face challenges, as transforming unstructured text into a simplified graph structure may result in the loss of nuanced context.

Following the application of inclusion criteria and rigorous quality assessment, 20 studies were selected for qualitative synthesis. The subsequent section analyzes these studies, focusing on the definition, implementation, and evaluation of personalization within LLM- and RAG-based healthcare AI systems. Tables 5, 6 provide a comparative overview of the identified information retrieval methodologies. Given the limited availability of healthcare-specific sources at the time of writing, this review primarily prioritized domain-specific healthcare literature.

**Table 5:** Comparative overview of RAG-based information retrieval approaches.

Reference	Domain	Approach	Task	Strength	Limitations
Alghamdi and Mostafa (2024)	Healthcare	Domain-specific fine-tuned LLM integrating synthetic data augmentation, multilingual prompting, RAG & evidence-verification agents	Reliable healthcare chatbot	Combined fine-tuning, RAG validation, and evidence-based verification to improve reliability and multilingual healthcare support in a culturally specific setting.	Focused mainly on information delivery and chatbot reliability rather than patient-specific personalization. Also, the study did not include physician review or an analysis of clinician agreement. No latency or scalability metrics were reported.
Goyal et al. (2024)	Healthcare AI	Domain-specific healthcare LLM combining instruction fine-tuning, supervised fine-tuning, prompt engineering, and RAG for SOAP-note generation	Automated medical documentation from doctor–patient conversations	Demonstrates that smaller domain-specialized LLMs can outperform general-purpose models such as GPT-4 for medical note generation through targeted fine-tuning and retrieval integration. Emphasizes clinically relevant challenges including hallucination mitigation, terminology understanding, physician-preferred writing styles, and long-context medical conversations. It also highlights practical deployment considerations such as quantization and HIPAA-compliant deployment pipelines.	The work focuses primarily on documentation efficiency rather than true patient-specific personalization. Evaluation details remain limited, with no rigorous clinical validation, longitudinal patient adaptation, or safety-oriented benchmarking. The paper largely presents a system-development narrative instead of a comprehensive empirical evaluation, and retrieval is mainly used to improve context handling rather than individualized clinical reasoning.
Yuan et al. (2023)	Healthcare	Privacy-aware LLM framework combining LLM-based data augmentation, Chain-of-Thought (CoT) prompting, ClinicalBERT embeddings and RAG	Patient–trial matching using EHRs and clinical trial eligibility criteria	Improved semantic interoperability between EHRs and trial criteria, achieving reported gains in matching performance and generalizability while preserving patient privacy through desensitized augmentation.	Focused mainly on semantic matching and augmentation rather than clinical reasoning. Real-world clinical validation, explainability analysis, and user-safety evaluation were not reported.
Wang et al. (2025)	Healthcare	A framework integrating RAG, CoT, LLM judges, ROUGE, and NER-based evaluation	Evaluating questioning capabilities of LLM healthcare chains in patient conversations	Introduced one of the first systematic frameworks for evaluating question-generation quality in healthcare conversations, linking high-quality questioning to improved patient information elicitation across multiple LLM architectures and judges.	Focused on evaluation of questioning strategies rather than personalized healthcare delivery and governance and regulatory compliance deployment. No latency or throughput score reported.
Rani et al. (2024)	Healthcare Diabetes-focused	Knowledge graph-based RAG framework integrating keyword retrieval, vector retrieval, graph retrieval, topic-based chunking, and source-traceability mechanisms	Reliable and explainable diabetes-focused healthcare question answering	Proposed a hybrid Graph-RAG framework combining structured and unstructured retrieval strategies to improve contextual relevance, explainability, and factual accuracy in healthcare responses. Introduced topic-based chunking and source-traceability mechanisms. Improved ROUGE performance compared with traditional RAG approaches.	The framework was evaluated primarily on diabetes-related QA tasks using ROUGE-based metrics rather than real-world clinical decision-making scenarios. Focus on retrieval quality and explainability without addressing personalized treatment adaptation or clinical deployment validation.
Zhang et al. (2025)	Home Healthcare NLP	LLM-RAG pipeline integrating Omaha System terminology, embedding-based retrieval, vector databases, few-shot learning, and CoT prompting	Automated identification and mapping of health problems from clinician–patient conversations	RAG-enhanced LLMs can accurately map conversational healthcare data to standardized clinical terminology, achieving strong F1 scores while reducing manual documentation burden. The framework systematically evaluated embedding models, prompting strategies, and context-window configurations for home healthcare dialogue analysis.	Focused primarily on standardized terminology mapping and documentation support rather than personalized clinical reasoning. Evaluation was limited to a relatively small home-healthcare dataset and controlled experimental settings, leaving broader clinical generalizability.
Zhao et al. (2025)	Healthcare	The framework integrating RAG, KGs-elicited reasoning, hierarchical diagnostic KGs, proactive questioning, and EHR retrieval.	Accurate diagnosis, treatment recommendation, and follow-up question generation for healthcare copilots.	KG-enhanced RAG framework that improves diagnostic specificity and reduces misdiagnosis for diseases with similar manifestations. Combined structured diagnostic KGs, EHR retrieval, & reasoning-based follow-up questioning, strong performance public & private datasets.	The framework remains heavily dependent on knowledge graph quality, structured EHR, and complex retrieval pipelines. Real-world deployment, scalability in healthcare systems and clinical validation remain limited. Personalization is follow-up questioning.
Richard-Ojo et al. (2024)	Healthcare	RAG chatbot with foundation model (llama 2 on AWS Bedrock. Augmented with PDFs	Generating reliable medical knowledge responses & enhancing text quality	Showing reduces hallucinations and improves ROUGE and METEOR scores over non-RAG baselines, offering a concrete cloud healthcare prototype.	Focused on response quality and lacks in personalized delivery, patient safety and governance-compliance. No latency, throughput, scalability score reported
Patil et al. (2025)	Healthcare RAG systems	RAGMed framework integrating LLaMA3-8B, Pinecone vector databases, semantic retrieval, embedding-model comparison & RAG-Triad evaluation	Medical question answering, appointment scheduling, and clinical note summarization	Proposed a practical RAG-based healthcare assistant that combines semantic retrieval with grounded LLM generation. Compared embedding models using clinically meaningful RAG-Triad metrics. Improved contextual relevance & summarization quality with high dimensional embeddings.	The system remains an earlystage proof of concept evaluated primarily on public datasets and a limited number of benchmark queries. Real-world deployment testing remains limited.

**Table 6:** Comparative overview of LLM-based information retrieval approaches.

Reference	Domain	Approach	Task	Strength	Limitations
Reategui-Rivera et al. (2025)	Mental Health	GPT-4–based conversational agent integrating anti-stigma educational modules, curated knowledge bases and prompt-guided interactions	Reducing mental illness stigma among healthcare providers through educational chatbot interactions	Combined evidence-based stigma-reduction frameworks with LLM conversational capabilities to deliver scalable, interactive educational interventions. Demonstrated strong performance in empathy generation, structured educational delivery, and realistic scenario generation.	Relied primarily on simulated evaluations and expert scoring. The framework focused on educational support with moderate performance in stigma detection. Scalability discussed but no numerical metric.
Shool et al. (2025)	Healthcare LLM evaluation	Large-scale systematic review of LLM evaluation methodologies	Analysis of healthcare LLM benchmarking and assessment practices	Provides comprehensive overview of 761 healthcare LLM evaluation studies and highlights the lack of standardized clinical evaluation frameworks	Primarily descriptive review: does not establish unified evaluation standards or fully address deployment-level personalization and safety validation
Zhou et al. (2025)	Healthcare	LLM-based automated analysis of physician–patient conversations using Phi-3.5	Identification of missed clinical information during primary-care encounters	Focused on improving patient safety. Demonstrates clinically grounded LLM deployment for improving communication quality and patient safety with strong identification accuracy.	Limited dataset size and restricted clinical scope; lacks in deployment evaluation. Trust and governance-compliance discussed and no score reported for computational & inference.
Ghosh et al. (2024)	Medical Summarization	Framework integrating CLIP, GPT-3.5, ImageBind-based filtration, and multimodal knowledge grounding	Medical question summarization using textual queries and medical images	A framework combining visual symptom cues with LLM-based contextual reasoning. Demonstrated improved factual recall, reduced hallucination, and better clinically relevant summaries through multimodal integration.	Primarily focused on summarization quality. It relied heavily on zero-shot prompting and simulated evaluation and real-world deployment safety remain insufficiently validated.
Vatsal et al. (2026)	Healthcare	Seven-dimensional empirical taxonomy for evaluating LLM-based healthcare agents.	Systematic evaluation and categorization of LLM-based agent frameworks	The framework provided structured empirical analysis of planning, reasoning, safety and clinical-task capabilities while identifying major gaps in adaptation, drift mitigation, and compliance readiness.	Not introducing a deployable clinical system or personalized. Scalability, latency, reliability, ethics, safety as open challenges and no measured.
Kilinc et al. (2025)	Generative AI in medicine	BERTopic-based bibliometric and topic-modeling analysis	Analysis of research trends in healthcare LLM applications	Provides large-scale overview of dominant themes, emerging trends, and application areas in medical generative AI research	Primarily descriptive trend analysis; does not evaluate clinical effectiveness, personalization quality, deployment real-world, or healthcare safety outcomes
Feng et al. (2023)	Healthcare LLM Application	Exploratory evaluation of ChatGPT	Assessing the feasibility, strengths, and risks of ChatGPT in healthcare and medical research	Provided practical evaluations of ChatGPT in healthcare, highlighting strengths in summarization and structured clinical-note generation. Critically examined ethical risks, hallucinations, misinformation generation, plagiarism, and misuse in medical research workflow	Primarily exploratory and qualitative, relying on prompt-based demonstrations rather than systematic benchmark evaluation or real-world clinical validation. Lacked standardized evaluation metrics. Safety discussed as concern; no safety metric.
Chekuri et al. (2025)	EHR messaging	LLM-based patient message classification and workflow support	Identifying patient questions in MyChart communication	Demonstrates practical LLM integration into real healthcare communication workflows	Focuses primarily on workflow optimization rather than examined clinical validation or evaluation & safety-aware reasoning such as ethical risks.
Genovese et al. (2026)	Healthcare LLM Evaluation	LLM-as-a-judge evaluation analysis.	Automated assessment of healthcare AI outputs	Critically evaluates scalability and potential utility of automated LLM-based healthcare evaluation	Highlights risk of unreliable clinical judgment, hallucinated evaluation, and insufficient clinician oversight; automated metrics may not reflect true clinical correctness or patient safety.
Furini et al. (2024)	Healthcare Chatbots	LLM-based healthcare chatbot communication framework using personality analysis, disease-context adaptation, Likert-scale evaluation, and AI-based clustering	Personalized healthcare communication and chatbot language adaptation	Demonstrated that effective chatbot communication requires combining both personality traits and disease context rather than relying on a one-size-fits-all strategy. Also, showed that AI-based clustering can improve personalization by identifying distinct communication preferences.	Relied on a relatively small participant sample and questionnaire-based evaluation rather than real-world clinical deployment. Personalization in this study was limited to communication style adaptation without incorporating clinical reasoning, or adaptive therapeutic decision-making.
Croxford et al. (2025)	Clinical Summarization	LLM-as-a-Judge framework using the validated PDSQI-9 instrument with zero-shot, few-shot, SFT, DPO, and multi-agent evaluation strategies	Automated evaluation of AI-generated clinical multi-document summaries from EHRs	Introduced a scalable automated evaluation framework for clinical summarization with strong agreement to human evaluators. GPT-o3-mini achieved high inter-rater reliability while substantially reducing evaluation time and cost. Also, Analyzed multi-agent evaluation workflows in HIPAA-compliant clinical environments.	Focused primarily on summarization evaluation rather than personalized clinical reasoning or direct patient-care support. Validation was limited to summarization tasks from specific healthcare systems, while broader clinical deployment, external validation, and generalization to other healthcare NLP tasks remain limited.

## 4 Taxonomy and synthesis of personalization in healthcare AI

The reviewed studies reveal that personalization in healthcare AI is implemented through multiple strategies, ranging from retrieval of patient-specific context to parameter adaptation and hybrid retrieval-generation frameworks. To clarify these approaches, the literature is organized according to the underlying personalization mechanism rather than by publication chronology.

### 4.1 Healthcare LLM applications

Recent studies have explored various healthcare chatbot implementations. For example, the Med-Pal chatbot (Elangovan et al., 2025) was introduced to assist users with medication inquiries. This chatbot system emphasizes generating patient-appropriate and safety-aware responses, positioning it as a valuable tool for health communication. It was developed using a curated dataset of medication-related questions and answers and evaluated across five lightweight LLMs with up to 7 billion parameters. Another notable implementation described in (Badlani et al., 2021) presents a multilingual chatbot with text-to-speech and speech-to-text capabilities. This chatbot system is designed to diagnose diseases based on user-reported symptoms and employs cosine similarity and TF-IDF, along with machine learning algorithms, to improve response accuracy. Similarly, the chatbot proposed in (Athota et al., 2020) uses AI and NLP technologies to diagnose diseases and offer basic health information, incorporating n-grams, TF-IDF, and cosine similarity for text processing and response generation. The primary objective of these systems is to reduce costs and assist users with minor concerns. However, these systems do not fully guarantee protection against clinically incorrect or irrelevant outputs, including hallucinations, which can undermine user trust and the validity of responses. Moreover, the generic advice offered by such systems may not always be clinically actionable, highlighting the need for improved safeguards and mitigating strategies. Cloud-based generative AI services have recently emerged as a promising solution for developing human-centered healthcare AI chatbot applications. These cloud services offer integrated tools for fine-tuning, RAG, and access to foundation models through unified APIs, enabling secure deployment and customization without direct infrastructure management. However, our experiments in Section 5 indicate that this approach lacks comprehensive evaluation of context relevance, answer faithfulness, and personalization. In addition, it raises open challenges related to privacy protection and cost efficiency. These concerns are particularly critical in healthcare settings, where real-world applications must protect sensitive information and prevent unintended data exposure. Consequently, this highlights the pressing need for systematic benchmarking frameworks for evaluating LLM- and RAG-based systems.

### 4.2 Challenges and limitations: privacy

In discussions about implementing AI systems in healthcare, several challenges arise, including ethical considerations, data privacy, and concerns about the correctness or incorrectness of AI-generated outcomes. These challenges necessitate careful examination of how AI systems influence patient decision-making and clinical care pathways. Such concerns form the foundation for Moral AI, which addresses both the technical reliability of AI healthcare systems and the ethical implications of incorporating AI into decision-making and question-answering processes. Moral responsibility is closely related to accountability for decisions and their subsequent consequences. Experts highlight that both moral accountability and safety assurance remain continuing challenges for complex AI systems, particularly in critical medical-care contexts (Habli et al., 2020). When clinicians lack direct and deliberate control over recommendations introduced by AI systems, it becomes necessary to engage AI developers and system safety experts in evaluating moral accountability. Ensuring safety is essential for both medical providers and patients, and accountability should be appropriately balanced in the design of AI healthcare system (Achter, 2013; Aveling et al., 2016). There is growing consensus that factors such as the extent of weakened human control, the patient safety risks, and levels of epistemic uncertainty must be carefully assessed to determine what is considered acceptably safe and morally justifiable prior to deploying digital healthcare system. For example, robotic-assisted laparoscopic surgery or automated dialysis machines operate under the direct supervision by medical professionals. Consequently, it is essential to proactively collect data and real-world experiences from the clinical use of AI systems by quantifying morally relevant effects of reliance on AI healthcare systems and assessing how clinical practice is influenced by the machine-generated recommendations.

In scenarios where AI healthcare systems participate in question answering or decision-making, clinicians may no longer retain direct control over the recommendations or decisions produced (Oshana, 2004), making it difficult to fully understand how input data are translated into outputs. Therefore, AI healthcare system should function as decision-support tools under oversight of qualified medical professionals, who remain responsible for determining the most appropriate course of action.

Despite rapid advances in AI-driven healthcare chatbots, many AI systems still fail to achieve meaningful personalization when interacting with patient data. For instance, a recent study (Zode et al., 2024) proposed an AI-powered chatbot integrated with electronic healthcare records (EHR) to enhance data security and provide access to medical advice. However, we observed that retrieved patient information was not effectively incorporated into the conversational context many question-answering (QA) benchmarks. Using a structured EHR dataset focused on diabetes-related test results, we found that although insulin test values were successfully retrieved and presented to the user, the chatbot system remained limited in delivering user-specific personalization or clinically meaningful responses to diabetes-related queries. Furthermore, the chatbot system’s ability to handle questions unrelated to the patient’s current condition remains uncertain and requires further evaluation. Additionally, the retrieval of personal health data is strictly limited to individual user records, which further constrains the system’s capacity for broader contextual reasoning and adaptive personalization.

In this review, we distinguish contextual adaptation from true clinical personalization. Retrieval grounding alone is not considered full personalization unless it incorporates patient-specific context, longitudinal history, preferences, or clinician-validated decision support.

### 4.3 Personalization strategies in AI healthcare systems

This section presents a synthesis of the main findings from the 20 reviewed studies. In this review, we distinguish contextual adaptation from true clinical personalization. While retrieval grounding and communication-style adjustment can improve response relevance, true personalization requires the use of patient-specific information, such as medical history, preferences, individual clinical needs, longitudinal context, or clinician-validated decision support. Retrieval augmentation integrates external knowledge with LLMs without modifying the model’s parameters. It combines a retriever component with LLMs to enhance generation quality and factual grounding. There are two approaches for obtaining relevant documents. In the first approach, documents are retrieved from an external corpus, such as Wikipedia or domain-specific databases. In the second approach, the LLM itself is employed to generate retrieval queries or candidate documents. Tables 5, 6 provide comparative overviews of information retrieval approaches based on RAG-based and LLM-only methods, respectively.

#### 4.3.1 Retriever-based personalization

Relevant documents are retrieved from an external corpus using sparse retrieval techniques such as Term Frequency-Inverse Document Frequency (TF-IDF) and Best Match 25 (BM25), which integrates document length normalization to provide more refined relevance scoring. While sparse approaches are robust for exact keyword matching, they often fail when relevance relies on contextual understanding rather than lexical overlap. An alternative is a Dense Passage Retrieval (DPR), in which queries and documents are projected into continuous vector spaces by neural network encoders, enabling retrieval based on semantic similarity. These dense methods have shown strong performance across many question-answering (QA) benchmarks and are integrated with sparse approaches in hybrid hierarchical retrieval frameworks, allowing complementary strength to be leveraged (Arivazhagan et al., 2023). There is a study developed a retrieval-augmented healthcare chatbot using Amazon Bedrock to address healthcare-related user queries through the integration of external knowledge retrieval and large language model generation. The study demonstrates the practical feasibility of deploying healthcare-oriented RAG systems using commercially available cloud infrastructure and highlights the potential of retrieval augmentation to improve response relevance and factual grounding. However, the work is primarily implementation-focused and provides limited evidence regarding clinical effectiveness, patient outcomes, or healthcare-specific personalization. Furthermore, the framework does not incorporate patient context, clinician evaluation, or comprehensive safety assessment. These limitations illustrate a common challenge in healthcare chatbot research, where deployment feasibility is often demonstrated before rigorous evaluation of personalization, safety, and real-world clinical impact. Although the system partially evaluates effectiveness by comparing the generated text against a reference text, additional metrics are required to fully assess relevance, accuracy, and safety (Richard-Ojo et al., 2024). In conversational agent designed through multilingual medical guidance and reliable healthcare information delivery (Alghamdi and Mostafa, 2024) combined domain-specific fine-tuning of GPT-3.5 Turbo and Llama3 models with synthetic data augmentation, RAG, prompt engineering, and evidence-based verification mechanisms. Incorporated both real-world and synthetic healthcare question–answer pairs tailored to the medical challenges. Integrated a secondary AI verification agent trained on the HealthVer dataset to validate generated medical responses against supporting evidence, while the RAG module improved response reliability through retrieval from trusted healthcare sources, showed that synthetic data augmentation and RAG integration improved chatbot accuracy and semantic quality metrics, with the RAG module reportedly increasing accuracy by approximately 5%. Furthermore, despite extensive benchmark-based evaluation using ROUGE, BERTScore, and accuracy metrics, the study remains limited in terms of real-world clinical validation and safety auditing (Goyal et al., 2024) introduced a domain-specific healthcare LLM from doctor–patient conversations. The framework combined instruction fine-tuning, supervised fine-tuning on medical-note datasets, and RAG to improve medical documentation quality. The study addressed several healthcare-specific challenges, including hallucination mitigation, complex medical terminology understanding and long-context clinical conversations. They argued that smaller domain-adapted LLMs can outperform larger general-purpose systems for specialized documentation tasks. However, the framework primarily focused on workflow automation rather than clinically grounded personalization, as it did not incorporate individualized treatment adaptation or patient-specific reasoning. Similarly, (Yuan et al., 2023) proposed a privacy-aware LLM framework for patient–trial matching that uses LLM-based data augmentation to improve compatibility between Electronic Health Records (EHRs) and clinical trial eligibility criteria. The framework leveraged Chain-of-Thought (CoT) prompting, desensitized patient data, and semantic-preserving augmentation techniques to generate enriched trial criteria while protecting sensitive patient information using ClinicalBERT embeddings. The study demonstrated improved patient-criteria and patient-trial matching performance, achieving a reported 7.32% average performance improvement and 12.12% gain in generalizability over baseline approaches. However, the framework primarily improves semantic matching and data augmentation rather than delivering true patient-specific personalization. The study relies mainly on experimental benchmark evaluation leaving concerns regarding safety or clinical validation of LLMs. Unlike traditional healthcare QA systems that primarily focus on answer generation (Wang et al., 2025) emphasized the importance of question formulation for obtaining clinically relevant patient information. The framework integrated multiple LLM architectures, including RAG and CoT, while combining LLM-judge interrogation metrics with traditional NLP measures such as ROUGE and Named Entity Recognition (NER)-based evaluation. Experimental results demonstrated that advanced reflective and CoT-based chains generated more relevant, specific, and informative questions, leading to improved patient-information elicitation across multiple benchmark datasets and LLM judges. However, the framework remains focused on evaluating conversational questioning quality rather than delivering clinically grounded personalization or real-world diagnostic reasoning. Furthermore, reliance on LLM-based evaluation metrics introduces potential bias and limits assessment of clinical safety. Enhances RAG through knowledge graph (KG)-elicited reasoning for clinical decision support (Zhao et al., 2025) proposed a healthcare copilot framework systematically constructs hierarchical diagnostic knowledge graphs from Electronic Health Records (EHRs) and integrates them with retrieved patient records to improve diagnostic specificity, treatment recommendation generation, and follow-up questioning. This framework explicitly models diagnostic differences between diseases with similar manifestations, enabling more accurate reasoning and reduced diagnostic ambiguity. With both the public dataset and a private chronic pain diagnostic dataset demonstrated outperformed several RAG approaches in diagnostic accuracy and specificity while also improving proactive follow-up question generation. The work is particularly significant because it combines structured medical reasoning, retrieval augmentation, and personalized diagnostic interaction within a unified architecture. However, this framework remains highly dependent on curated diagnostic knowledge graphs, structured EHR infrastructure, and complex retrieval pipelines, which may limit scalability in healthcare systems. Similarly, (Rani et al., 2024) designed a framework to improve the reliability, explainability, and contextual relevance of diabetes-focused healthcare LLMs. The framework integrated three retrieval strategies—keyword retrieval, vector retrieval, and graph retrieval—within a unified retrieval pipeline supported by topic-based chunking and source-traceability mechanisms. To improve factual consistency and reduce hallucinations, they constructed a validated diabetes-specific knowledge base from trusted healthcare resources, which was then transformed into a structured knowledge graph using LLM-driven graph construction techniques. The evaluation demonstrated that the proposed Graph-RAG framework substantially outperformed traditional RAG approaches in ROUGE-based evaluation metrics. However, the evaluates retrieval quality and response overlap rather than patient personalization. Also relied mainly on benchmark-style diabetes question-answering tasks and ROUGE metrics. Another proposed an LLM-RAG framework for automatically identifying health problems from home healthcare conversations and mapping them to standardized clinical terminology. By combining retrieval-augmented generation with terminology standardization, the framework facilitates the transformation of unstructured conversational data into structured clinical information, improving documentation consistency and interoperability across healthcare systems. The study demonstrates the potential of LLM-RAG systems to support real-world healthcare workflows by reducing manual documentation burden and enhancing information extraction accuracy. However, the framework focuses primarily on problem identification and terminology normalization rather than patient-centered personalization, as it does not incorporate longitudinal patient modeling, adaptive clinical reasoning, or large-scale clinician-centered evaluation. This highlights a broader challenge in healthcare AI, where improvements in clinical documentation and information extraction do not necessarily translate into meaningful personalization of patient care (Zhang et al., 2025). In addition, Patil et al. introduced RAGMed, a retrieval-augmented medical AI assistant designed to support healthcare delivery through clinically grounded question answering and information retrieval. By integrating external medical knowledge with large language model generation, the framework aims to improve factual consistency and reduce reliance on static parametric knowledge. The study highlights the potential of retrieval-augmented approaches to enhance healthcare information access and support clinical decision-making tasks. However, the framework primarily focuses on improving information retrieval and response generation rather than achieving true patient-centered personalization. In addition, evidence regarding large-scale clinical deployment, clinician-guided validation, and patient-safety assessment remains limited. This reflects a broader trend in healthcare RAG systems, where improvements in factual grounding and retrieval quality often outpace advances in personalized clinical reasoning and longitudinal patient adaptation (Patil et al., 2025).

Overall, the reviewed studies employ diverse evaluation methods to assess generation quality, contextual relevance and addressing data privacy and ethical concerns, which are essential factors in the healthcare field.

#### 4.3.2 Hybrid personalization

In generative retrieval approach, LLMs generates relevant documents or passages directly rather than retrieving them from an external corpus. For example, (Shi et al., 2024) proposed a Personalized Medical Language Model (PMLM) that combines recommendation systems and reinforcement learning to adapt personalized prompts using patient preferences and behavioral patterns. Although the framework demonstrated promising individualized responses in obstetrics and gynecology settings, and the system still falls short of clinically grounded personalization and safety-aware evaluation remain limited restricting assessment of the framework’s robustness and real-world clinical reliability. In medical summarization framework (Ghosh et al., 2024) proposed a multimodal that integrates CLIP, GPT-3.5, and ImageBind-based knowledge filtration to generate medically aware summaries from both textual patient queries and medical images. Combining healthcare questions with visual symptom representations. The proposed framework incorporated multimodal disorder identification, contextual medical knowledge generation, filtration of hallucinated content, and LLM-based summary generation to improve clinically relevant summarization. This improvements in factual recall, multimodal fact-capturing, and hallucination reduction compared with text-only baselines, highlighting the benefit of integrating visual symptom information into healthcare NLP workflows. Also, the system relies mainly on zero-shot prompting and controlled benchmark evaluation. The study primarily to personalization through multimodal contextualization, enabling visual symptom information to augment textual patient descriptions and improved medical question summarization quality, the evaluation focuses on summarization performance rather than clinical correctness, patient safety, or deployment considerations. No hallucination, latency, trust, or clinician-validation metrics were reported. In Conversational agent designed to reduce mental illness stigma among healthcare providers through interactive educational interventions (Reategui-Rivera et al., 2025) proposed Stigma Educational Bot 1.0, a GPT-4–based. The framework combined anti-stigma educational principles, curated knowledge resources, and prompt-guided conversational design to deliver scalable mental health education. The strong performance in educational content delivery, empathetic response generation, and realistic clinical scenario simulation, however, primarily functioned as an educational support tool rather than a personalized mental healthcare framework. Evaluation relied mainly on simulated tasks and expert assessment, limiting validation of long-term behavioral impact. The feasibility of ChatGPT in healthcare (Cascella et al., 2023) demonstrated that ChatGPT could effectively summarize medical information, organize structured clinical notes, generate scientific-style text, and provide context-aware responses for healthcare communication tasks, highlighted the model’s ability to improve outputs iteratively through conversational prompting and its usefulness in simplifying technical medical information for patients and clinicians. The study emphasized several important limitations, including hallucinated reasoning, inaccurate causal interpretation, ethical concerns, plagiarism risks, misinformation generation, and misuse for fabricated scientific outputs. The work represents an important early exploratory assessment of healthcare LLM capabilities rather than a clinically validated healthcare AI framework. Furthermore, the evaluation remained largely qualitative and prompt-driven without standardized benchmarking, real patient-care validation, or rigorous assessment of robustness and safety.

For more focus on personalized healthcare communication in conversational strategies for LLM-based healthcare chatbots (Furini et al., 2024) analyzed how personality traits and disease types of influence user preferences toward factual and medical communication styles using a Likert-scale questionnaire across multiple healthcare scenarios. It showed that neither personality traits nor disease categories alone were sufficient for effective personalization; instead, combining both factors provided communication adaptation. Also, they applied AI-based clustering methods to identify distinct user groups and demonstrated that communication preferences differed across clusters, highlights the importance of psychologically informed personalization strategies in healthcare chatbot design prior to system prototyping and deployment. However, the study focuses on communication-style adaptation rather than patient-specific healthcare reasoning. The evaluation relied on questionnaire-based user perception analysis with a relatively small participant population, limiting assessment of real-world clinical effectiveness and long-term healthcare impact. Another explored (Chekuri et al., 2025) the use of LLMs to identify and categorize patient questions within MyChart messaging systems to improve healthcare communication efficiency and workflow support. The framework demonstrated the potential of LLMs to assist with large-scale patient-provider communication management in real clinical environments. However, the approach primarily focused on message classification and workflow optimization rather than clinically grounded personalization, as it did not incorporate longitudinal patient modeling, clinician-guided adaptation, or safety-aware reasoning. This illustrates a broader trend in healthcare LLM deployment, where operational efficiency improvements often outpace advances in true patient-centered personalization (Shool et al., 2025) conducted a large-scale systematic review of 761 studies evaluating large language models in clinical medicine, examining benchmarking methodologies, evaluation metrics, specialties, and model architectures. It demonstrated that healthcare LLM evaluations remain heavily dominated by general-domain models and inconsistent assessment practices, with accuracy serving as the most frequently reported metric. Importantly, the study highlighted the lack of standardized healthcare evaluation frameworks and the limited representation of medically specialized LLMs. These findings reinforce broader concerns regarding the reliability and consistency of current healthcare LLM evaluation methodologies, particularly when benchmark-driven performance metrics are interpreted as indicators of clinical readiness or meaningful personalization (Zhou et al., 2025) developed an LLM-based framework using Phi-3.5 to automatically identify missed information during physician–patient communication in primary-care clinical encounters. By analyzing real consultation transcripts, the framework demonstrated strong performance in detecting missing diagnostic information while also emphasizing the importance of physician empathy and communication clarity for patient satisfaction. This work is grounded in real clinical interactions and patient-safety concerns. However, the framework remains limited by its relatively small dataset and narrow clinical scope. Another study (Kilinc et al., 2025) analyzed emerging research trends in medical generative AI using BERTopic-based topic modeling and bibliometric analysis of 3,941 publications related to LLM applications in medicine. They identified dominant themes in areas such as radiology, ophthalmology, mental health, clinical decision support, and patient-centered care, demonstrating the rapidly expanding role of generative AI across healthcare domains. It remains primarily descriptive and trend-oriented, without evaluating clinical effectiveness, patient-centered personalization, or deployment-level safety considerations. This underscores the need for more advanced approaches capable of effectively integrating and processing detailed, domain-specific information to ensure reliable and accurate answers that support personalization.

Overall, these studies address contextual quality, personalization capability, and privacy preservation, highlighting both progress and remaining challenges in deploying real-world personalized healthcare AI applications.

#### 4.3.3 Evaluation and governance

Several recent studies have shifted attention from system development toward evaluation, governance, and deployment readiness. Rather than proposing new personalization architectures, these works examine how healthcare AI systems should be validated, monitored, and governed before clinical deployment. For healthcare LLM-as-a-Judge (LaaJ) systems (Li et al., 2026) proposed the MedJUDGE framework for risk-stratified evaluation governance. The study systematically analyzed validation practices, bias testing, deployment readiness, and safety limitations across healthcare LaaJ applications, highlighting major concerns such as model monoculture, limited human oversight, and insufficient fairness evaluation. To address these issues, the authors introduced the MedJUDGE framework, which organizes healthcare AI evaluation around validity, safety, and accountability pillars combined with clinical risk-tier classification. However, the work remains primarily governance- and evaluation-oriented rather than focused on patient-specific personalization and lack real-world clinical validation. In the same way, (Bedi et al., 2024) proposed a structured five-axis evaluation framework that categorized studies according to evaluation data type, healthcare task, NLP task, dimension of evaluation, and medical specialty. Their analysis demonstrated that current healthcare LLM evaluations remain heavily concentrated on medical question answering and exam-style benchmarking, with 84.2% of studies focusing on question answering tasks and 95.4% emphasizing accuracy as the primary evaluation dimension. In contrast, important dimensions such as fairness, robustness and deployment considerations were rarely assessed. Further highlighted the uneven distribution of evaluations across medical specialties, with fields such as medical genetics and physical medicine receiving minimal attention. They provide strong methodological synthesis and establish an important healthcare-specific evaluation taxonomy; however, it remains primarily an evaluative survey rather than a clinically validated deployment framework. Also, the study identifies evaluation gaps and governance challenges but does not experimentally implement standardized benchmarking pipelines or real-world healthcare deployment strategies. In addition, (Croxford et al., 2025) an automated LLM-as-a-Judge framework for evaluating AI-generated clinical multi-document summaries using the validated Provider Documentation Summarization Quality Instrument (PDSQI-9). The study systematically evaluated multiple prompting and training strategies, including zero-shot, few-shot, supervised fine-tuning (SFT), direct preference optimization (DPO), and multi-agent evaluation workflows across both reasoning and non-reasoning LLMs. It demonstrated that GPT-o3-mini achieved strong agreement with expert physician evaluators, reaching an intraclass correlation coefficient (ICC) of 0.818 while reducing evaluation time from approximately 600 s for human review to 22 s per automated evaluation. The work is particularly significant because it addressed a major bottleneck in healthcare AI deployment by introducing scalable and cost-efficient evaluation of clinical summarization quality within HIPAA-compliant environments. Also highlighted the superiority of reasoning-based models over standard generative models for evaluating clinical attributes such as synthesis, organization, and thoroughness. However, it remains focused on automated evaluation infrastructure rather than personalized patient-specific healthcare reasoning and the validation was largely restricted to summarization tasks using data from limited healthcare.

Examined the emerging role of LLM-as-a-Judge systems in healthcare, (Genovese et al., 2026) focusing on their potential to automate evaluation, fact-checking, and quality assessment for clinical AI applications. It discussed how LLM judges are increasingly being integrated into healthcare workflows for tasks such as clinical summarization assessment, documentation validation, benchmark evaluation, and automated reasoning verification. They highlighted several advantages of LLM evaluators, including scalability, rapid assessment, reduced annotation burden, and strong agreement with expert reviewers in narrowly defined tasks. At the same time, the study provided a detailed analysis of major risks associated with healthcare LLM judges, including hallucination propagation, evaluator monoculture, demographic bias, shared-ignorance failures between judge and generator models, and the lack of transparent accountability mechanisms. They further emphasized that healthcare evaluation systems require governance-aware design, auditable reasoning processes, fairness testing, and clinician-centered oversight to ensure trustworthy deployment. However, it remains primarily conceptual and governance-oriented rather than experimentally validated in large-scale clinical environments. The study emphasizes that automated evaluation metrics alone may not adequately capture clinical correctness or patient safety, reinforcing broader concerns regarding the reliability of benchmark-driven healthcare LLM assessment. In analysis highlighted important architectural trends, (Vatsal et al., 2026) including the dominance of multi-agent frameworks and retrieval-grounded clinical support systems, while also identifying substantial weaknesses in drift mitigation, event-triggered activation, and long-term adaptive learning. The work remains primarily a taxonomy and evaluation-oriented survey rather than a clinically validated healthcare framework, rely on literature-level categorization and qualitative scoring rather than prospective deployment studies, limiting direct assessment of personalization capability and operational robustness in the healthcare field. On agentic paradigms for LLM-enabled healthcare communication (Zhi et al., 2025) focusing on the transition from traditional reactive LLM systems toward autonomous clinical agents capable of planning, memory management, collaboration, and self-evolution. The study introduced a novel taxonomy structured around two orthogonal dimensions: knowledge source and agency objective, leading to four major paradigms including Latent Space Clinician, Grounded Synthesizer, Emergent Planner, and Verifiable Workflow Automator. This work emphasized the underlying cognitive architecture and operational mechanisms governing clinical agents. They systematically analyzed core components and reviewed implementation frameworks, benchmark datasets, and evaluation metrics relevant to clinical dialogue systems while discussing major challenges including hallucination, safety, interpretability, and trustworthy deployment. However, the work remains largely conceptual and architectural, with limited empirical validation in real-world clinical workflows and does not provide extensive evidence regarding large-scale deployment robustness, longitudinal patient impact, or regulatory feasibility in operational healthcare settings.

#### 4.3.4 Comparative analysis of RAG-based and LLM-based approaches across studies

**Figure 9:**
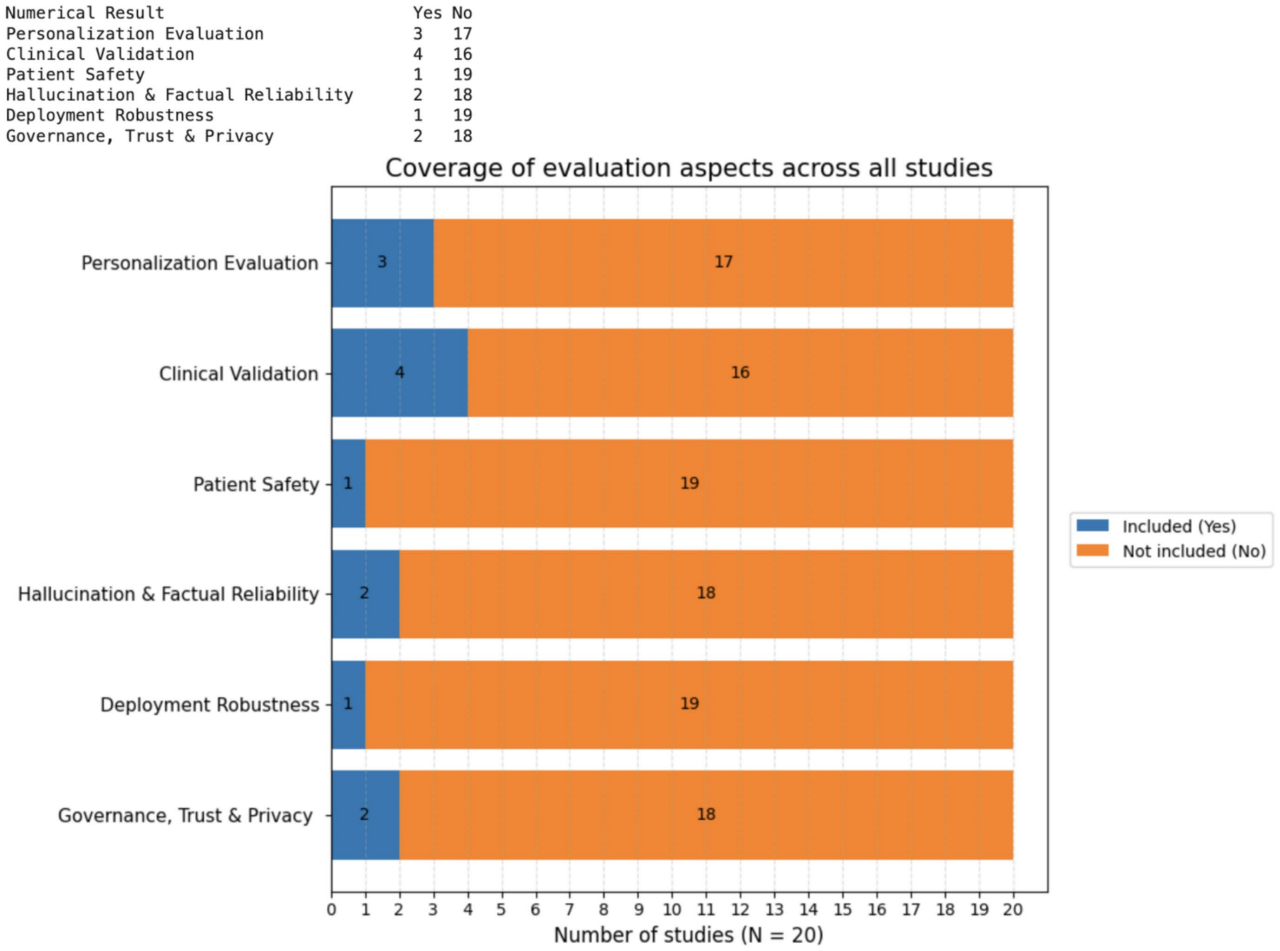
Distribution metrics evaluation results among studies.

This section synthesizes findings from the comparative evaluation of RAG-based systems and fine-tuned LLMs, revealing significant gaps in the assessment of hallucination, patient safety, and deployment performance. Figure 9 summarizes the coverage of these evaluation aspects across the reviewed studies.

Table 7 summarizes the diverse evaluation strategies adopted across the reviewed studies, highlighting substantial variation in the metrics, benchmarks, and validation procedures used to assess healthcare-oriented LLM and RAG systems. These studies employed a combination of automated metrics, expert assessment, information extraction measures, and retrieval-grounding evaluations. The comparison also highlights substantial differences in how personalization is defined, implemented, and evaluated across healthcare studies.

**Table 7:** Cross-study comparison of evaluation metrics reported across the reviewed studies, shown in sub tables (a)–(e).

(a) Wang et al. (2025)—Multi-metric evaluation of LLM healthcare chains							
Model-metric	RAG	RAGdefault	RAGreflection	RAG_reflection_cot	RAG_reflection_cot_sc	ReAct	Hardcoded
GPT-4o Specificity	7.38	6.80	7.98	7.68	7.28	5.52	5.30
GPT-4o Usefulness	8.27	7.95	8.82	8.54	8.12	5.59	5.02
GPT-4o Relevance	8.19	7.89	8.72	8.43	8.03	5.46	4.63
GPT-4o Coverage	4.54	4.56	5.39	5.11	4.90	3.63	3.69
GPT-4o Fluency	9.36	9.01	9.47	9.33	9.12	8.44	6.70
GPT-3.5 Specificity	7.18	6.30	7.84	7.50	7.02	4.59	5.26
GPT-3.5 Usefulness	7.99	7.20	8.53	8.21	7.89	5.75	5.18
GPT-3.5 Relevance	8.98	8.59	9.18	9.05	8.82	5.68	4.83
GPT-3.5 Coverage	5.18	4.94	5.77	5.53	5.24	4.34	4.38
GPT-3.5 Fluency	9.08	9.00	9.23	9.13	9.00	7.92	6.80
Claude Specificity	7.31	5.93	7.88	7.57	7.12	3.65	4.30
Claude Usefulness	7.96	6.07	8.38	8.13	7.86	4.70	5.31
Claude Relevance	8.88	6.69	9.28	9.07	8.79	3.83	5.41
Claude Coverage	4.89	5.73	5.64	5.35	5.09	2.29	3.70
Claude Fluency	9.06	8.24	9.17	9.12	8.98	7.84	6.57

(b) Zhou et al. (2025)—Identification of missed information during the diagnostic process				
Variables	Accuracy	Precision	Recall	F1-score
Daily habits	0.7703	0.9216	0.7833	0.8468
Medical history	0.9595	0.9595	1.0000	0.9793
Physical exam	0.9730	0.9863	0.9863	0.9863
Average	0.9009	0.9558	0.9232	0.9375

(c) Reategui-Rivera et al. (2025)—(1) Identification of stigma in simulated clinical interactions and (2) Generated testimony evaluation									
	Stigma level	Recall	Precision	F1-score		Testimony type	Consistency	Perceived empathy	Overall task completion
(1)	Very obvious	0.39	0.62	0.48	(2)	Patient 1	4.5 ± 0.7	4.5 ± 0.7	4.5 ± 0.7
	Subtle	0.49	0.60	0.53		Patient 2	4.5 ± 0.7	4.0 ± 1.4	4.5 ± 0.7
	Very subtle	0.40	0.88	0.54		Health provider 1	4.5 ± 0.7	4.0 ± 1.4	4.0 ± 1.4
						Health provider 2	5.0 ± 0.0	4.5 ± 0.7	4.5 ± 0.7

(d) Patil et al. (2025)—Average RAG-TRIAD scores across 18 queries				
Query/metric	Embedding model	Answer relevance	Context relevance	Groundedness
Average across 18 queries	GTE-Large	0.72	0.70	0.47
	all-MiniLM-L6-v2	0.53	0.61	0.31

(e) Chekuri et al. (2025)—Comparative evaluation of question detection methods for patient messages						
Model	Dataset	Recall	Precision	Specificity	Accuracy	F1-score
SyntaQD	Set A	0.95	0.83	0.90	0.92	0.88
Mixtral-8x7B	Set A	0.99	0.66	0.73	0.82	0.79
GPT-4	Set A	0.80	0.92	0.97	0.91	0.86
BERT	Set A	0.27	0.81	0.97	0.71	0.40
SyntaQD	Set B	0.76	0.81	0.88	0.83	0.78
Mixtral-8x7B	Set B	0.99	0.71	0.73	0.83	0.83
GPT-4	Set B	0.74	0.93	0.96	0.87	0.82
BERT	Set B	0.21	0.81	0.97	0.66	0.34

The quantitative findings in Table 7 were synthesized across six evaluation dimensions: personalization, hallucination and factual reliability, clinical validation, patient safety, deployment robustness and governance, trustworthiness and privacy.

##### 4.3.4.1 Personalization evaluation

Most reviewed studies assessed personalization through retrieval quality, information elicitation, contextual adaptation, or extraction of patient-specific information. Wang et al. (2025) evaluated healthcare questioning chains using LLM judges and demonstrated that questions rated as more relevant, useful, and specific elicited responses that more closely matched the ground-truth patient information, indicating improved information personalization and retrieval grounding. Patil et al. (2025) assessed retrieval quality through answer relevance and context relevance, showing that GTE-Large outperformed all-MiniLM-L6-v2 across all evaluation dimensions, with answer relevance increasing from 0.53 to 0.72. Similarly, Zhang et al. (2025) evaluated automated identification of health problems and symptom classification, achieving precision, recall, and F1-scores of 0.90 using GPT-4o-mini, demonstrating the ability of LLM-based systems to accurately capture clinically relevant patient information. Several additional studies incorporated contextual information, retrieval augmentation to improve personalization (Zhao et al., 2025; Yuan et al., 2023; Ghosh et al., 2024); however, most systems focused on retrieval-grounded adaptation rather than patient preferences, longitudinal health histories, or individualized clinical. These findings suggest that current healthcare personalization is primarily achieved through improved retrieval and contextual grounding rather than comprehensive patient-specific reasoning.

##### 4.3.4.2 Hallucination and factual reliability

Many studies identified hallucination mitigation as an important objective, yet relatively few reported quantitative measures of hallucination or factual consistency. Several studies proposed mechanisms to improve grounding and reduce unsupported outputs, including knowledge graphs, retrieval augmentation, verification modules, and privacy-aware retrieval strategies (Zhao et al., 2025; Alghamdi and Mostafa, 2024; Yuan et al., 2023; Richard-Ojo et al., 2024; Rani et al., 2024; Zhang et al., 2025; Genovese et al., 2026). However, most evaluated these approaches qualitatively rather than through explicit hallucination rates or faithfulness scores. Among the studies reporting quantitative metrics, Wang et al. (2025) demonstrated that healthcare chains receiving higher relevance and usefulness scores produced responses that more closely matched ground-truth patient information. Similarly, Patil et al. (2025) evaluated groundedness using the RAG-Triad framework and found that GTE-Large achieved higher groundedness (0.47) than all-MiniLM-L6-v2 (0.31), indicating improved factual alignment between retrieved information and generated responses. These findings suggest that retrieval quality and grounding mechanisms are increasingly used as indirect measures of hallucination control, although standardized hallucination such as rates or score remain uncommon.

##### 4.3.4.3 Governance, trust, and privacy

Governance-related concerns, including privacy, transparency, fairness, bias and human oversight, were frequently discussed across the reviewed studies but were rarely evaluated quantitatively. Privacy-preserving architectures were proposed in (Alghamdi and Mostafa, 2024; Yuan et al., 2023) studies, although quantitative privacy metrics were generally absent. Study (Genovese et al., 2026) provided one of the strongest governance-focused discussions, emphasizing auditability, bias testing, human oversight, and appeal mechanisms. User trust and satisfaction were evaluated more directly in some studies. For example, (Alghamdi and Mostafa, 2024) reported that 68% of users were very satisfied and 20% were satisfied with chatbot responses, corresponding to approximately 88% positive satisfaction. Likewise, Zhou et al. (2025) found that empathetic communication (OR = 3.609) and clear explanations (OR = 5.051) were strongly associated with higher patient satisfaction. Reategui-Rivera et al. (2025) further reported perceived empathy scores of up to 4.5/5, demonstrating the importance of communication quality and trust-building in healthcare AI systems.

##### 4.3.4.4 Clinical validation and patient safety

Only a limited number of studies performed explicit clinical validation using expert assessment or clinically grounded benchmarks. Reategui-Rivera et al. (2025) employed mental health experts to evaluate outputs and establish gold-standard annotations for stigma-related tasks. Croxford et al. (2025) demonstrated that an LLM-as-a-Judge achieved strong agreement with expert evaluators (ICC = 0.818), while maintaining reliability across a secondary task (ICC = 0.710), suggesting that automated evaluation may effectively supplement human review. Zhou et al. (2025) focused directly on identifying missed information during clinical encounters, achieving average accuracy, precision, recall, and F1-scores of 0.9009, 0.9558, 0.9232, and 0.9375, respectively. Notably, physicians missed daily-habit information in approximately 19% of encounters, highlighting potential patient-safety implications associated with incomplete clinical documentation. Nevertheless, quantitative assessments of patient safety, adverse clinical outcomes, and harmful recommendations remain limited across the current literature.

##### 4.3.4.5 Deployment robustness, efficiency, and scalability

Deployment robustness was frequently discussed, particularly regarding scalability, latency, throughput, and computational efficiency, although quantitative measurements were uncommon. Patil et al. (2025) described lower-latency embedding models and millisecond-scale retrieval but did not report comprehensive latency benchmarks. Similarly, studies (Rani et al., 2024; Zhang et al., 2025) discussed scalability conceptually without providing throughput or response-time measurements. Among the reviewed studies, Croxford et al. (2025) provided the strongest quantitative evidence for deployment efficiency, showing that GPT-o3-mini completed evaluations in 22 s compared with approximately 600 s for human evaluators. Chekuri et al. (2025) compared multiple approaches for question detection in real-world patient messaging, demonstrating trade-offs between recall, precision, and accuracy that are important for large-scale deployment. Collectively, these findings indicate growing attention to deployment considerations, although systematic evaluation of latency, throughput, and scalability remains limited.

Overall, the reviewed studies demonstrate increasing emphasis on retrieval grounding, expert validation, and trust-oriented evaluation. However, evaluation practices remain highly varied. While factual reliability, satisfaction, and retrieval quality are commonly reported, quantitative assessments of hallucination rates, patient safety outcomes, privacy preservation, and deployment performance remain relatively rare. This highlights the need for healthcare-specific evaluation frameworks that assess clinical validity, safety, reliability, trustworthiness, and operational performance. The quantitative findings summarized in Table 7 provide limited evidence that some RAG-based approaches and fine-tuned LLM systems can improve retrieval grounding, task performance, and evaluation efficiency. However, direct assessments of hallucination rates, patient safety outcomes, and deployment robustness remain uncommon. Consequently, the current literature provides only limited evidence regarding the factors most critical for real-world healthcare deployment.

**Figure 10:**
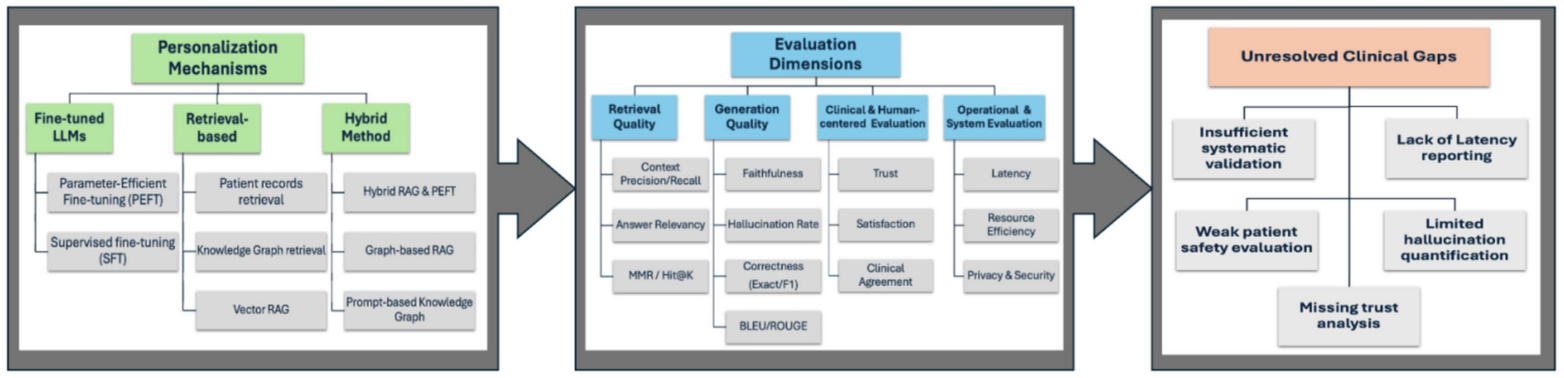
Taxonomy of personalization strategies, evaluation dimensions, and unresolved clinical gaps in healthcare AI.

To provide a high-level synthesis of personalization strategies, evaluation dimensions, and unresolved challenges identified across the reviewed studies, Figure 10 presents a unified taxonomy linking implementation approaches to evaluation practices and remaining clinical gaps.

Overall, the reviewed studies show that healthcare RAG and LLM systems are moving toward more grounded, explainable, and workflow-aware designs. However, most systems still provide contextual adaptation rather than true longitudinal personalization, and evaluation remains dominated by benchmark metrics rather than prospective clinical validation, patient safety outcomes, and deployment robustness. Personalization remains limited, with most studies relying on retrieval grounding rather than patient-specific adaptation. Clinical validation is infrequently reported, with only a subset of studies involving clinician evaluation. Patient safety evaluation is rarely performed, despite the healthcare setting. Hallucination assessment remains inconsistent, with few studies providing quantitative measurements. Deployment metrics such as latency and efficiency are seldom reported, limiting real-world applicability.

## 5 Illustrative case study: evaluation limitations in hybrid rag

To complement the literature synthesis, we include a brief illustrative case study. This section presents a brief illustrative case study to highlight limitations of current evaluation metrics used to quantify performance in hybrid RAG systems. These metrics are typically categorized into two groups: retriever-based approaches (Yu et al., 2024; Maryamah et al., 2024) and answer-generator-based approaches (Ip, 2025; Ragas, 2025). To ensure clarity and reproducibility, we briefly define the evaluation metrics used in this case study, following standard RAG evaluation frameworks (Yu et al., 2024; Maryamah et al., 2024; Furini et al., 2024; Ip, 2025; Ragas, 2025). Contextual recall measures whether the retrieved documents contain all information required to answer the query, while contextual precision evaluates the proportion of retrieved content that is relevant. Contextual relevancy assesses the semantic alignment between the retrieved context and the query. For generation, answer relevancy measures how well the generated response addresses the query, and faithfulness evaluates whether the response is grounded in the retrieved context without introducing unsupported information. All metrics are normalized to a score range of 0 to 1, where higher values indicate better performance, and were computed using semantic similarity and LLM-based evaluation in our pipeline. To illustrate these limitations, we conducted simulation-based experiments using the Medical Question Answering Dataset (MedQuAD; Ben Abacha and Demner-Fushman, 2019), a publicly available resource curated and reviewed by licensed medical professionals. The reference answers used in this case study are derived directly from the MedQuAD dataset, rather than newly annotated by the authors, ensuring consistency with clinically curated source material. While this case study evaluates retrieval grounding and answer generation within a medical question-answering setting, it does not assess full healthcare personalization, which would require integration of patient-specific longitudinal context, comorbidity-aware reasoning, and individualized clinical decision support. This case study is not intended for statistical generalization; therefore, formal statistical measures such as confidence intervals or variance analysis were not included, as the goal is to illustrate evaluation limitations rather than benchmark performance.

### 5.1 Retriever evaluation

Retriever evaluation assesses whether the retrieval component can locate relevant, accurate, and sufficient context information to high-quality generation. This process employs a variation of the question-answer generation to derive metrics such as contextual recall, contextual precision and contextual relevancy, which are used to evaluate top-k retrieval performance and embedding models. The evaluations examines whether the retrieved context includes all required information to generate an optimal output and whether the retrieved passages are correctly ranked, with the most relevant context appearing first for a given query. Together, these factors influence the quality of the final generated output. n our experiment for this type of evaluation, we employ a variation of the question–answer generation to derive metric scores, as shown in Table 8. Most queries achieve perfect or near-perfect scores (e.g., 1.00) across all three metrics—contextual recall, precision, and relevancy—with only minor variations in specific instances. The high scores likely reflect the constrained and highly curated nature of the evaluated question–answer pairs rather than robust evidence of clinically realistic retrieval performance. However, this evaluation approach has important limitations. Since this method only retrieves existing information and cannot compensate for an incomplete external corpus, which highlights the questionable adequacy of this evaluation workflow for response quality in retrieval and raises concerns whether it efficiently accesses related document topics and provides truly relevant information. Each retrieval component was evaluated based on whether the retrieval context included all required information to generate the optimal output, and whether the ranking prioritized the most relevant content. These played a role in the final response output score as shown below.

**Table 8:** Simulation retriever result.

Number of query	Contextual relevancy	Contextual precision	Contextual recall
1	1.00	1.00	1.00
2	0.95	1.00	0.85714
3	1.00	1.00	1.00
4	0.625	1.00	1.00
5	0.999	1.00	1.00

These results highlight a limitation of metric-based evaluation in simplified medical QA settings, where strong scores may coexist with limited evidence of personalization or real-world safety.

### 5.2 Answer-generator-based evaluation

While the generator-answer decides how effectively that context is used to generate useful responses. The process for generating answers starts by providing the relevant documents and the query to the LLM such as answer relevancy and faithfulness. In answer relevancy, the metric measures how well the generated response matches the given input, while in faithfulness, the metric measures whether the generated response includes any hallucination relative to the retrieval context and ranges from 0 to 1, where a higher score represents a better consistency. In our experiments, we used the same dataset and category to conduct this type of evaluation, and the results shown in Table 9 indicate high faithfulness scores (1.00) across all five topic queries, with answer relevancy ranging from 0.58 to 1.00, which shows differences in variation depending on the query topic. However, this type of evaluation can successfully retrieve specific information; however, its effectiveness depends on whether the query involves complex or simple medical topics and whether domain-specific information is missing.

**Table 9:** Simulation answer generation results.

Number of query	Answer relevancy	Faithfulness
1	0.9285714	1.00
2	0.72	1.00
3	0.882352	1.00
4	0.583334	1.00
5	1.00	1.00

The case study highlights a limitation of current evaluation frameworks rather than providing evidence of true personalization or safe deployment readiness. These findings reinforce broader concerns observed across the reviewed literature and motivate discussion of evaluation gaps.

## 6 Discussion

Across the reviewed studies, personalization in healthcare AI is primarily implemented through retrieval-based mechanisms rather than true patient-specific modeling. Most systems rely on retrieving relevant external knowledge at inference time rather than incorporating longitudinal patient context such as medical history or treatment trajectories. Our comparative overview highlights that information retrieval techniques have advanced significantly across sparse, dense, hybrid, and knowledge graph paradigms. Sparse retrieval offers high precision and controllability; dense and hybrid methods capture semantic similarity; and knowledge graph integration adds explainability. These gains come with trade-offs, including embedding compute and memory cost, latency from multi-stage retrieval and re-ranking, privacy exposure, and domain adaptability issues. Despite these advances, retrieval-based systems remain vulnerable to factual errors, hallucinations, and domain-specific inconsistencies, requiring additional mechanisms to improve reliability and trustworthiness. Using LLMs as generative evaluators is attractive for coverage and speed; however, in healthcare it remains costly, prompt-sensitive, and imperfectly aligned with expert clinical judgments, as noted in the previous section. This misalignment is particularly critical in healthcare settings, where incorrect or misleading responses may directly affect patient understanding and safety. The illustrative case study further supports the observation that current evaluation metrics for both retrieval and generation exhibit limitations related to query complexity and corpus completeness. These limitations lead to discrepancies in correctness, clarity, and clinical salience, particularly for domain-specific medical information, indicating that existing evaluation practices require further improvement.

Overall, progress in retrieval is improving relevance, faithfulness, and explainability; however, relatively few studies directly evaluate hallucination and factual reliability metrics, patient safety outcomes, regulatory compliance, personalization evaluation, deployment robustness, or clinical validation, despite their importance for real-world healthcare deployment. Furthermore, most current systems focus on single-condition queries, whereas real-world healthcare scenarios require personalization that accounts for multiple chronic conditions, medication interactions, and evolving patient states. Systems that integrate retrieval design with multi-layer evaluation are better positioned to deliver reliable healthcare AI. However, such approaches remain insufficient without clinically grounded validation.

These findings highlight several unresolved challenges in healthcare personalization, underscoring the need for further research and deployment. Building on these observations, the following section outlines key directions for future research.

## 7 Future direction

**Figure 11:**
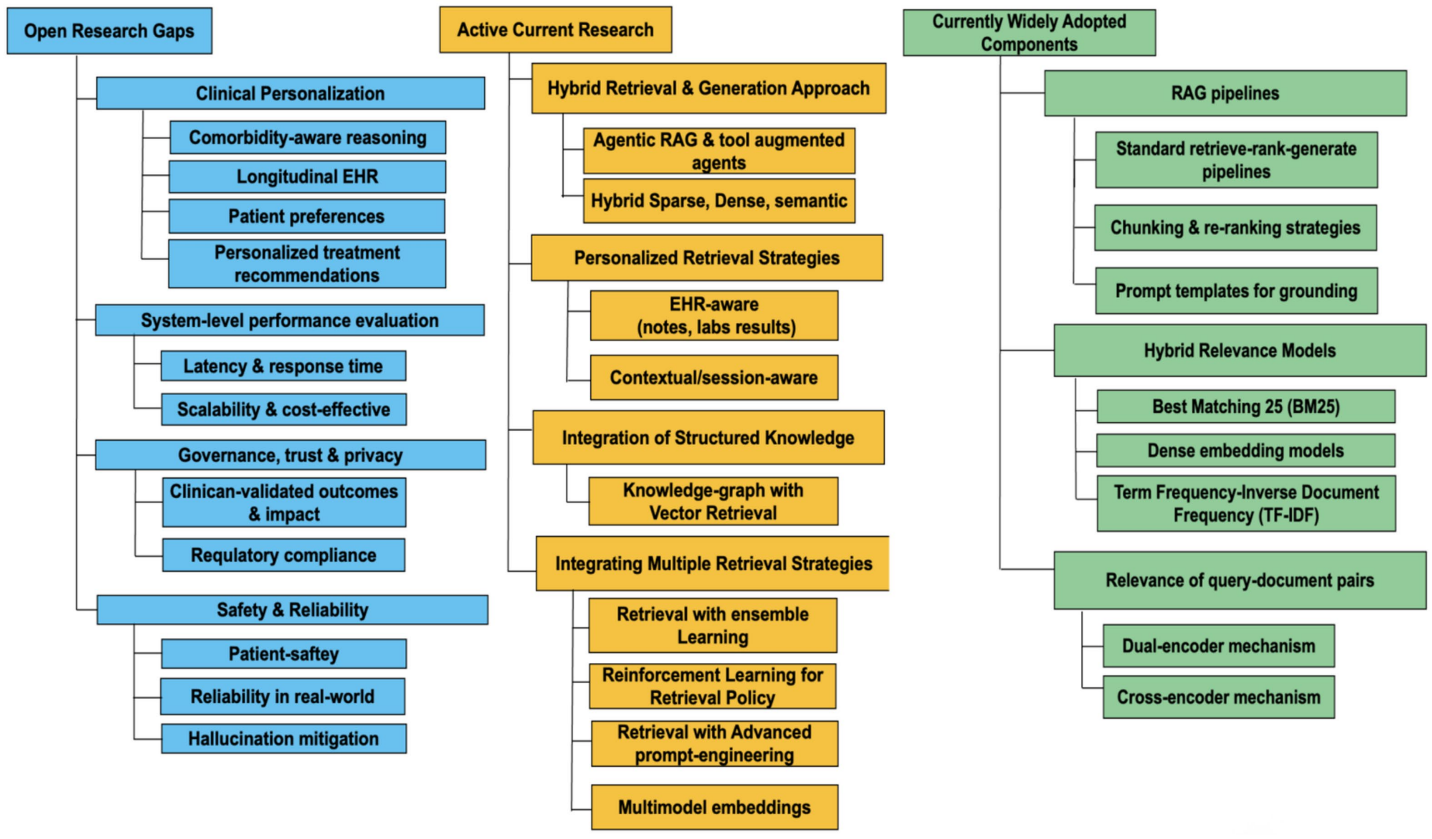
Roadmap of future research directions for personalization in RAG- and LLM-based healthcare AI systems.

Combining complementary retrieval and generative models remains an important direction for developing more advanced RAG-based and LLM-based personalization systems in healthcare. Figure 11 categorizes implemented solutions, existing gaps, and active research directions. Future work should focus more specifically on clinically validated, privacy-preserving, and patient-centered personalization rather than general model scaling or vendor-specific model improvements.

### 7.1 Standardized definitions of personalization

A major challenge in the current literature is the lack of a consistent definition of personalization. Many studies describe retrieval relevance as personalization, while true healthcare personalization should incorporate patient-specific history, preferences, and clinical context. Establishing standardized definitions and evaluation criteria is essential for improving comparability across studies.

### 7.2 Longitudinal EHR-aware personalization

Future systems should move beyond single-query evaluation and support longitudinal Electronic Health Record (EHR)-aware personalization. This allows models to adapt to patient history, evolving conditions, treatment progression, and long-term care, leading to more clinically meaningful personalized responses.

### 7.3 Comorbidity-aware retrieval

Healthcare retrieval should also support comorbidity-aware reasoning rather than focusing only on single-disease retrieval. Patients often present with multiple chronic conditions, and future retrieval systems must account for interactions between diagnoses, medications, and treatment plans.

### 7.4 Patient safety and clinician-validated evaluation

Model performance evaluation metrics should be further investigated in terms of robustness, fairness, bias, toxicity, uncertainty, and efficiency. Clinician-validated evaluation is necessary to ensure clinical relevance, safety, trustworthiness, and explainability. This will provide a more comprehensive and clinically aligned assessment of personalized healthcare AI systems.

### 7.5 Knowledge graph and hybrid retrieval integration

Integrating structured knowledge from knowledge graphs with unstructured text retrieval remains a promising direction. Knowledge graphs provide semantic relationships and contextual understanding, while vector retrieval improves semantic matching. Hybrid retrieval strategies combining sparse, dense, and graph-based retrieval can improve contextual relevance, explainability, and response quality (Wang et al., 2018).

### 7.6 Privacy, trust, and governance in LLM-/RAG-based personalization

At the same time, personalization using patient data raises major privacy, security, and governance concerns including regulatory frameworks such as HIPAA and GDPR that govern the use and sharing of sensitive patient data. Personal health information collected by clinicians is a critical asset for diagnosis and treatment and must be rigorously protected in AI-integrated systems using personalized retrieval (Barreda et al., 2025; Bhatt and Vaghela, 2024). Patients remain concerned about exposure of sensitive data, while hospitals are cautious about integrating internal repositories with AI systems due to ethical obligations and consent requirements (Fan et al., 2024; Kim et al., 2023).

### 7.7 Hallucination and factual reliability evaluation

Relatively few studies directly measure hallucination rates, groundedness and factual consistency. Future research should establish standardized evaluation protocols for assessing hallucinations and their clinical impact, particularly in high-risk healthcare settings.

### 7.8 Deployment robustness and real-world healthcare performance

While many studies evaluated retrieval quality, answer relevance, and factual grounding, few assessed deployment robustness in real-world healthcare settings. Metrics such as latency, scalability, computational efficiency, and workflow integration were rarely reported. Future research should therefore incorporate system-level evaluations that measure response time, scalability under increasing workloads, and integration with clinical workflows. Such assessments are necessary to ensure that AI systems can be deployed reliably and effectively in routine healthcare practice.

These future directions emphasize that personalization in healthcare AI must be approached as both a technical and clinical challenge, requiring systems that are interpretable, safe, regulation- compliant, and genuinely responsive to patient-specific context.

## 8 Conclusion

This survey examined personalization in healthcare AI through both LLM-only and RAG-enhanced paradigms, presenting a comparative view of retrieval and generation strategies. Our analysis suggests that personalization remains promising but still immature, as the literature is fragmented across datasets, evaluation methods, and application domains, and lacks alignment with clinically validated decision-support requirements. While advances in sparse, dense, hybrid, and knowledge-graph retrieval have improved relevance, grounding, and explainability, retrieval alone does not constitute true personalization. In many studies, personalization is achieved through retrieval of relevant information rather than deeper patient-specific adaptation involving longitudinal history, preferences, and clinical context. These improvements also introduce challenges related to computational cost, latency, privacy, and domain transferability. Although LLM-based evaluation frameworks are gaining attention, they remain costly, prompt-sensitive, and only partially aligned with expert clinical judgment. Our MedQuAD-based case study shows that high metric scores do not always mean clinically correct or reliable results. These findings highlight a fundamental gap between current evaluation practices and the requirements of clinically reliable, deployment-ready healthcare AI systems. Most current systems perform retrieval-grounded contextual adaptation rather than true patient-centered personalization. Furthermore, relatively few studies directly evaluate hallucination rates, patient safety outcomes, clinician validation, or deployment robustness, despite their importance for real-world healthcare implementation. Addressing this gap will require healthcare-specific benchmarks, clearer definitions of personalization, and evaluation frameworks that incorporate clinical validation, safety and real-world deployment constraints.
